# CIZ1-F, an alternatively spliced variant of the DNA replication protein CIZ1 with distinct expression and localisation, is overrepresented in early stage common solid tumours

**DOI:** 10.1080/15384101.2018.1526600

**Published:** 2018-10-06

**Authors:** Dorian R.A. Swarts, Emma R. Stewart, Gillian S. Higgins, Dawn Coverley

**Affiliations:** Department of Biology, The University of York, York, UK

**Keywords:** CDKN1A-interacting zinc finger protein 1, nuclear matrix, alternative splicing

## Abstract

CIZ1 promotes cyclin-dependent DNA replication and resides in sub-nuclear foci that are part of the protein nuclear matrix (NM), and in RNA assemblies that are enriched at the inactive X chromosome (Xi) in female cells. It is subjected to alternative splicing, with specific variants implicated in adult and pediatric cancers. CIZ1-F is characterized by a frame shift that results from splicing exons 8–12 leading to inclusion of a short alternative reading frame (ARF), excluding the previously characterized C-terminal NM anchor domain. Here, we apply a set of novel variant-selective molecular tools targeted to the ARF to profile the expression of CIZ1-F at both transcript and protein levels, with focus on its relationship with the RNA-dependent and -independent fractions of the NM. Unlike full-length CIZ1, CIZ1-F does not accumulate at Xi, though like full-length CIZ1 it does resist extraction with DNase. Notably, CIZ1-F is sensitive to RNase identifying it as part of the RNA-fraction of the NM. In quiescent cells *CIZ1*-F transcript expression is suppressed and CIZ1-F protein is excluded from the nucleus, with re-expression not observed until the second cell cycle after exit from quiescence. Importantly, *CIZ1*-F is over-expressed in common solid tumors including colon and breast, pronounced in early stage but not highly-proliferative late stage tumors. Moreover, expression was significantly higher in hormone receptor negative breast tumors than receptor positive tumors. Together these data show that CIZ1-F is expressed in proliferating cells in an unusual cell cycle-dependent manner, and suggest that it may have potential as a tumor biomarker.

## Introduction

**Figure 1.**
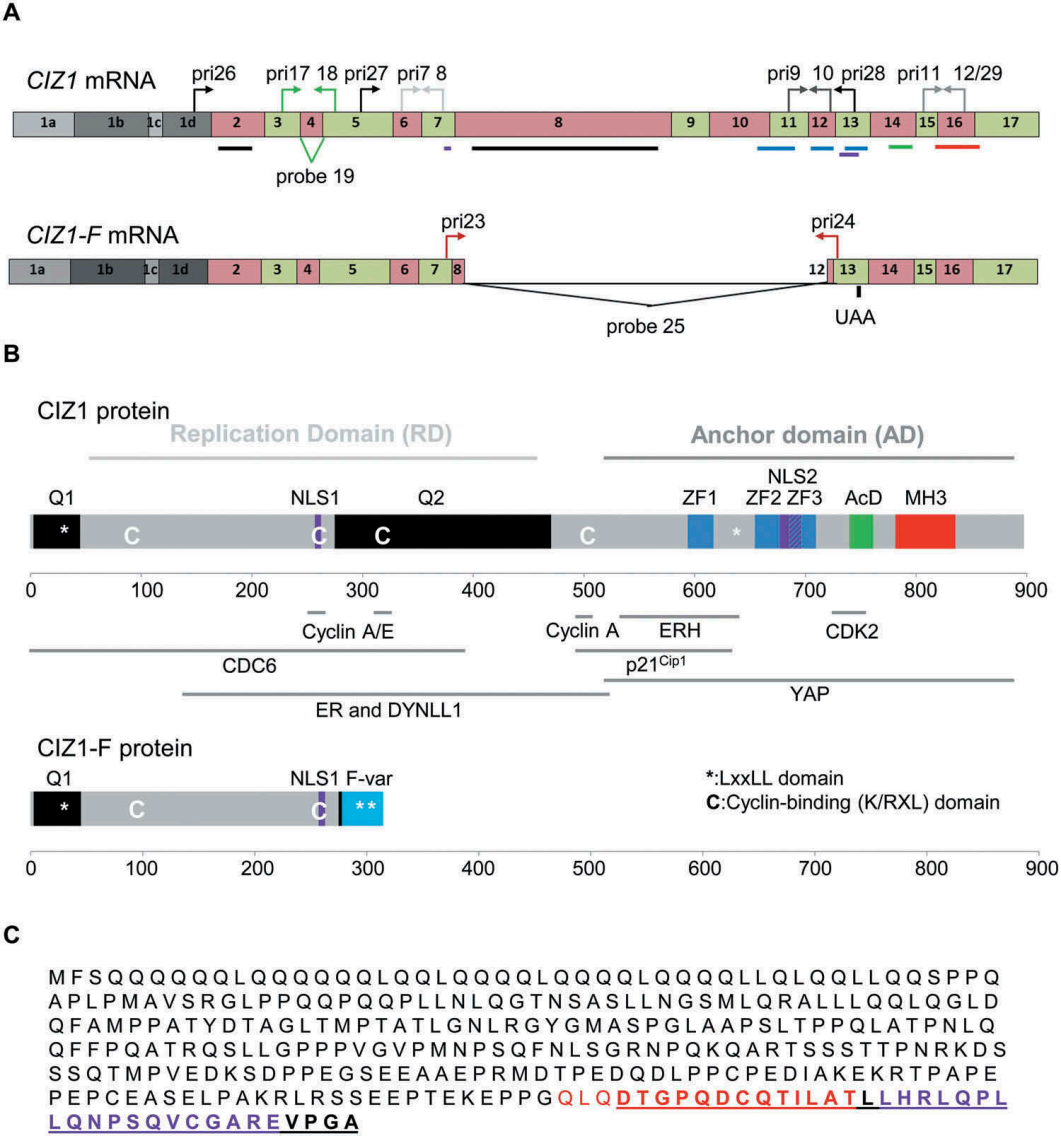
Human CIZ1 and variant CIZ1-F. (A) Exonic sequence in messenger RNA of full-length and *CIZ1*-F, with location of primers (pri) and Taqman probes indicated. Sequences are provided in Supplementary Table 2. Lines indicate the location of sequences that encode the protein domains illustrated in (B). Four alternative exon 1s are indicated; *CIZ1*-F has been detected in combination with exons 1b, 1c, and 1d. (B) Full-length and CIZ1-F protein showing annotated protein domains including glutamine-rich regions (Q), nuclear localization signals (NLS), zinc finger domains (ZF), matrin-3 domain (MH3), and acidic domain (AcD). Characterized functional domains and interaction sites for full-length CIZ1 are also shown, including replication domain (RD) which contains all sequences required for replication activity [6], and anchor domain (AD) which contains sequences that are sufficient to mediate attachment to the nuclear matrix [8]. The CIZ1-F specific sequence, encoded by an alternative reading frame (ARF) of exon 12–13, is highlighted in light blue. Also shown are the location of K/RXL cyclin-binding motifs implicated in replication [7] (‘C’), LXXLL ER-interaction motifs (asterisks), and sequences reported for full-length CIZ1 to interact with CDC6 [18], cyclin-dependent kinase 2 (CDK2) [15], cyclins A and E [7], dynein light chain (DYNLL1) [15], estrogen-receptor (ER) [12], enhancer of rudimentary homolog (ERH) [42], p21 [16], and YAP [17]. Note that several of these interactions were discovered in mouse CIZ1, and that the reported interaction between ER and CIZ1 is not via a domain that includes LXXLL [12]. X-axis shows amino acid number. (C) Amino acid sequence of the CIZ1-F protein, with the sequence specified by its ARF bold and underlined. The peptides in red and purple were used to generate CIZ1-F-specific antibodies.

CDKN1A interacting zinc finger protein 1 (CIZ1) is subject to extensive alternative splicing to yield multiple transcript variants [1–5]. Functional analysis of discrete protein fragments has identified domains and regulatory sites in the N-terminal half of the protein that are involved in DNA replication [6,7] and interaction with cell cycle regulators [7], (Figure 1(a,b)). Separate sequences in the C-terminus of CIZ1 support interaction with nuclear structures, identifying a salt and DNase-resistant nuclear matrix (NM) interaction domain [8], which may restrict its function to specific sub-nuclear locations. Recently, we and others showed that CIZ1 localizes to the inactive X chromosome (Xi) and is required to ensure retention of Xist long non-coding RNA (lncRNA) at Xi in fibroblasts [9,10]. Alternative splicing affects both the replication domain (RD) and NM anchor domain (AD) of CIZ1, and several splicing events have been implicated in cancer, including mutation driven exclusion of exon 4 (CIZ1-Δ4) in Ewing’s Tumor cell lines [2] and exclusion of 24 nucleotides of exon 14 in lung cancer (CIZ1-B) [5]. A unique splice-junction epitope from CIZ1-B is a circulating biomarker for patients with small cell and non-small cell lung cancer [5,11]. CIZ1 has also been reported to be involved in the development of breast cancer [12], the most common type of cancer in women [13]. Alternative splicing of CIZ1 is implicated in other chronic diseases including upregulation of a form in which part of exon 8 is excluded (CIZ1-S) in Alzheimer’s disease [3], and in cervical dystonia where mutations in an exonic splicing enhancer may affect splicing patterns [14].

Despite functional studies that link full-length murine CIZ1 and embryonic variant murine CIZ1 (ECIZ1) with DNA replication [6,7], little is known about the function of other variants. Alternative splicing of *CIZ1* may have important consequences for the cell and is known to affect the sub-nuclear localization of the protein (Supplementary Table 1). For example, exclusion of exon 4 changes the sub-nuclear distribution of CIZ1 from focal to diffuse [2]. Thus alternative splicing of *CIZ1* may influence the spatial organization of DNA replication, and suggests that some CIZ1 splice variants have the potency to act as dominant-negative controllers of other variants [2].

We previously designed an exon-junction microarray that identified a number of novel variants of CIZ1, including one common cancer-associated *CIZ1*-variant characterized by a continuous deletion of part of exon 8, exon 9–11 and part of exon 12, here referred to as CIZ1-F (Figure 1(a)) [1]. CIZ1-F was initially identified in Ewing’s Tumor cell lines and found to be overexpressed in primary lung tumors but not matched normal tissues [1]. Here, we characterize CIZ1-F mRNA and protein expression patterns by exploiting a short unique peptide encoded by expression of an alternative reading frame (ARF) of exon 12–13. We show that CIZ1-F is specifically expressed in G1 phase following mitosis but not G1 phase following quiescence. Despite lacking the C-terminal NM-attachment domain [8] and several protein interaction sites that are important for replication function, CIZ1-F resists extraction with DNase1. It is however sensitive to RNase, which contrasts with the behavior of full-length CIZ1. Most importantly, *CIZ1*-F is overexpressed in early stage primary human tumors and is associated with ER-negative status in breast tumors.

## Results

Human *CIZ1*-F is characterized by an 1181 base pair deletion (c1038_2218del1181 in reference sequence NM_012127.2), which excludes part of exons 8 and 12 and all of exons 9–11 (Figure 1(a), Supplementary Figure 1(a)). The exon 8–12 junction causes a frame-shift in the *CIZ1* reading frame, leading to a premature translational stop codon in exon 13, despite the presence of downstream exons in *CIZ1*-F mRNA (Figure 1(a)). CIZ1-F protein therefore lacks previously characterized functional domains, including the MH3-domain which anchors CIZ1 to the NM [8], and cyclin-binding motifs through which CIZ1 interacts with cyclin A, and which are essential for its DNA replication activity (Figure 1(b)) [6,7]. Other characterized sites of interaction with CDK2, p21^cip1^ and YAP are also excluded [15–17], while interaction with CDC6 [18], ERα [12], and dynein light chain [15] might be retained. The interaction sites of the CIZ1 binding factors PDRG1 [19], SH3BP4 [20], and TCF4 [21] are not yet characterized. Importantly, the ARF created by the 8–12 junction encodes 37 amino acids of unique sequence not present in other forms of CIZ1, or in other proteins, and results in inclusion of two additional LXXLL estrogen receptor (ER)-interaction motifs (Figure 1(b,c)) [22]. Thus *CIZ1*-F transcript appears to encode a 315 amino acid protein with a unique C-terminal end. To study *CIZ1*-F mRNA expression we designed a *CIZ1*-F junction-specific gene expression probe (Supplementary Table 2, Supplementary Figure 1(b-c)). Initially, *CIZ1*-F mRNA levels were measured by qRT-PCR in a set of cell lines (Supplementary Figure 1(d)), revealing variable levels, with high expression in bladder and cervical cancer lines EJ and HeLa, as well as in breast cancer cell lines MCF-7, BT474 and MDA-MB-231. Evaluation of cultured tumor-derived cell lines showed that *CIZ1*-F transcript is elevated in MCF7 breast cancer cells compared to normal MCF-10A breast cells (Supplementary Figure 1(d)), and is in fact one of the main *CIZ1* PCR-products (Supplementary Figure 1(a)), identifying MCF7 cells as a model cell line to study the function of CIZ1-F in cell proliferation. However, despite the reported functional interaction between CIZ1 and ERα [12], we were unable to detect a reproducible effect of estrogen on the expression of *CIZ1* or *CIZ1*-F transcript in MCF7 cells (Supplementary Figure 1(e)).

### *CIZ1*-F protein is part of the RNA-dependent NM

In order to confirm that *CIZ1*-F transcript encodes a protein we generated an affinity-purified anti-peptide antibody against sequences in the unique C-terminal ARF of predicted CIZ1-F protein (Figure 1(c)). Specificity of the antibody was confirmed using ectopically expressed EGFP-full-length CIZ1 and EGFP-CIZ1-F for western blot and immunofluorescence applications (Supplementary Figure 2(a-b)). Further confirmation of the specificity of our CIZ1-F antibody was obtained using peptide blocking, which yielded two CIZ1-F-specific products of approximately 42kD and 71kD in total cell lysates (Supplementary Figure 2(c)). The 42kD monomeric CIZ1-F product is larger than the predicted molecular weight of CIZ1-F (34kD) but in agreement with the mobility of EGFP-CIZ1-F after subtraction of EGFP (45kD; Supplementary Figure 2(a)).

**Figure 2.**
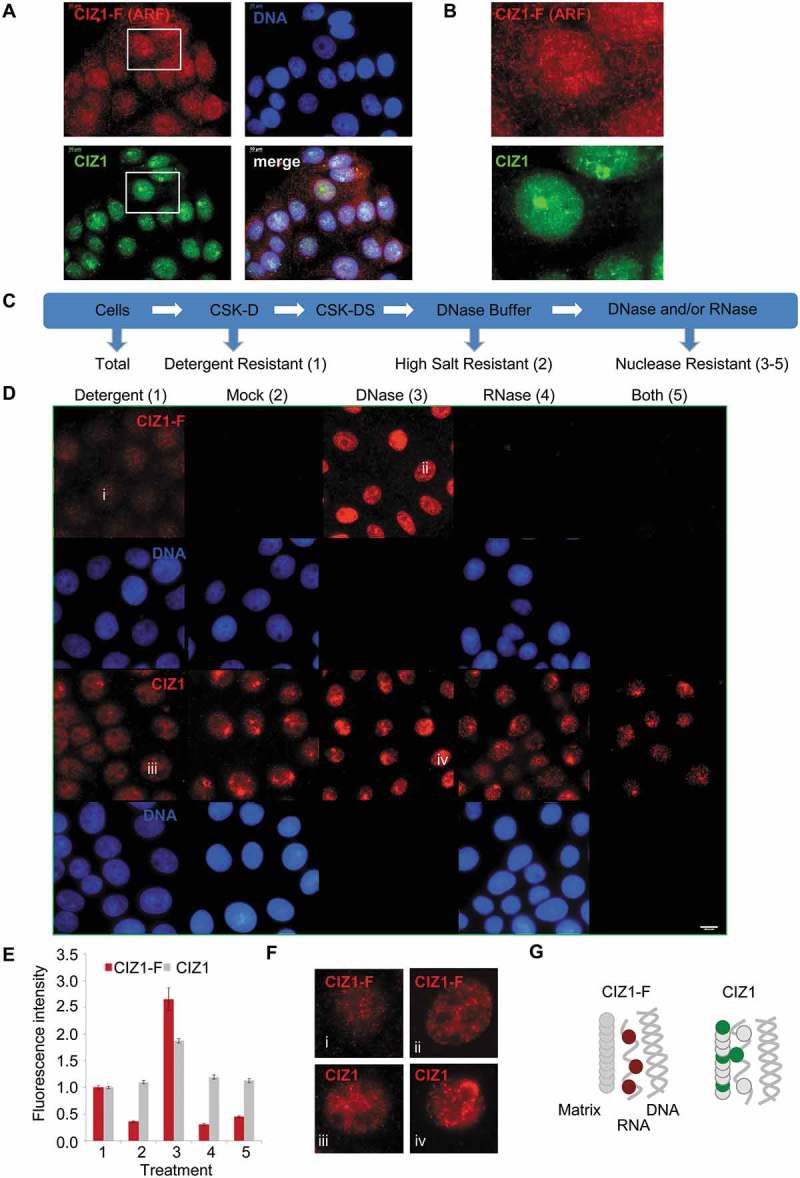
Nuclear CIZ1-F resists extraction of chromatin but not RNA. (A-B) CIZ1-F does not localize to the inactive X chromosome (Xi). Immunofluorescence with a monoclonal antibody for a peptide located in the CIZ1 anchor domain (AD; green) shows both diffuse nuclear expression and specific localization to a region previously shown to be the Xi [9]. Immunofluorescence with purified antibody raised to the CIZ1-F ARF (red) shows absence of Xi-localization. DNA is stained with Hoechst33258 (blue). Nuclei in white boxes are enlarged in (B). (C) Overview of nuclear matrix (NM) extraction procedure. MCF-7 cells were serially extracted with 1) detergent-containing cytoskeletal buffer (CSK), 2) detergent-containing CSK supplemented with 0.5 M NaCl (mock extraction), 3) DNase 1, or 4) RNase, or 5) both enzymes (see methods). (D) MCF-7 cells subjected to the treatments described under (C) were counterstained with Hoechst33258 to control for removal of chromatin (blue), and probed with purified polyclonal anti-CIZ1-F or replication domain (CIZ1-RD) antibody 1794 (red). CIZ1-RD resists all treatments, while CIZ1-F is sensitive to extraction of RNA, and is also dramatically revealed by removal of chromatin. Bar is 10 microns. (E) Quantification of the fluorescence intensities in (D), shown after subtraction of background signal and expressed relative to detergent treated cells. Number of cells quantified ≥ 70 per condition. Numbers refer to the treatment conditions listed above. (F) Enhanced and enlarged images of the indicated nuclei i-iv from (D), showing detergent and DNase-resistant nuclear fractions. (G) Interpretation of the data showing the dependency of CIZ1-F on RNA for nuclear retention, compared to full resistance of CIZ1-RD.

Since full-length CIZ1 localizes to the Xi and interacts with Xist lncRNA, we first determined the relationship between CIZ1-F and the Xi in MCF-7 cells. Full-length CIZ1, detected by a monoclonal antibody to a C-terminal epitope, clearly localizes to the Xi in MCF-7 cells, though in line with reports that Xi in breast cancer-derived cells is a less discrete (compact) entity than in ‘normal’ cells [23] Xi staining is markedly irregular, and also accompanied by the nucleus wide signal that is typical of CIZ1 [9]. In contrast, CIZ1-F shows a different pattern including both nuclear and cytoplasmic foci, but no enrichment at Xi (Figure 2(a-b)). We next examined the relationship of full-length CIZ1 and CIZ1-F with the NM. Full-length CIZ1 is part of the core protein NM (resistant to extraction with DNase and RNase), as evident by immunodetection of endogenous CIZ1 with RD-specific antibodies (Figure 2(c-f)). Analysis of attachment to this core protein NM can be recapitulated using EGFP-full-length CIZ1 and derived fragments, allowing the domain responsible for attachment to the NM to be located at the C-terminal end, and classified as ‘anchor domain’ or AD [8]. AD is missing from CIZ1-F, which would therefore not be expected to resist extraction. Importantly, under identical conditions to those used to profile endogenous CIZ1, the extraction profile for CIZ1-F indicates attachment to the RNA component of the NM (RNA-protein NM) because it resists extraction with DNase, but not when used in conjunction with RNase (Figure 2(c-f)). Notably, direct comparison of immunofluorescence signal in nuclei at different stages in the extraction process shows that the majority of the CIZ1-F epitope in the nucleus is in fact masked by chromatin (Figure 2(e)). The signal seen before extraction appears to be not the same population as that revealed when chromatin is removed, because pre-extraction signal is lost in mock treated nuclei and only revealed if DNase is also included in the reaction (Figure 2(e)). Again, despite the reported association between ERα and CIZ1 [12], we did not observe co-localization of CIZ1-F with nuclear ERα in immuno-detection experiments (Supplementary Figure 2(d)).

In conclusion, CIZ1-F resists extraction of chromatin and is therefore a NM protein, however it is not part of the core protein NM like full-length CIZ1 as it can be fully extracted by digestion of nuclear RNA, in the presence or absence of co-extraction of DNA, identifying it as part of the RNA-protein NM (Figure 2(g)). The differential extraction profiles of full-length CIZ1 and CIZ1-F epitopes suggest that they may have distinct functions within the NM.

### *CIZ1*-F expression in cycling and quiescent cells

**Figure 3.**
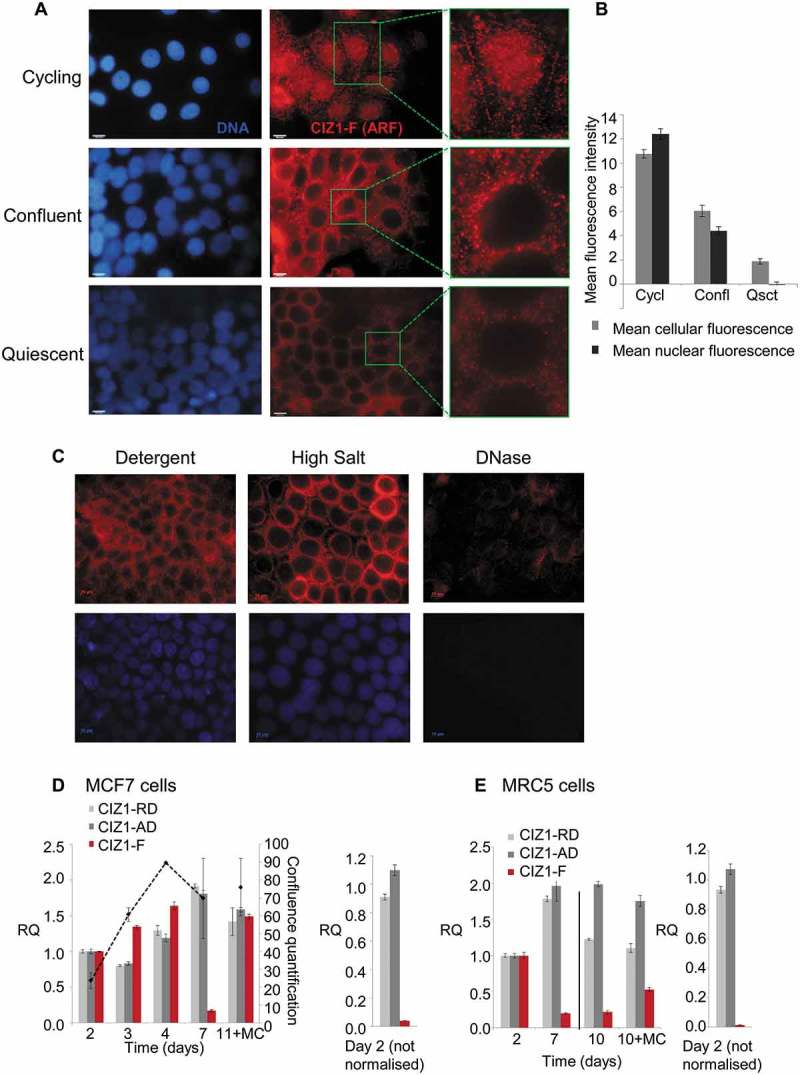
CIZ1-F transcript is suppressed and CIZ1-F protein is excluded from the nuclear matrix in quiescent cells. (A) Immunodetection of CIZ1-F in cycling (day 2), confluent (day 4) and quiescent (day 7 with medium change) populations of MCF-7 cells, after washing with detergent-cytoskeletal buffer to remove soluble protein. Enlarged images of example cells are shown and DAPI-stained images (left) show cell nuclei in the same fields. Bar is 10 microns. (B) Quantification of the average immunofluorescence signal of cells depicted in (A) after subtraction of background. Abbreviations: Cycl, cycling; Confl, confluent; Qsct, quiescent. (C) NM-extraction of quiescent MCF-7 cell populations showing no CIZ1-F on the NM after treatment with DNase. For details on the method see Figure 2(a). (D) Transcript levels in MCF-7 breast epithelial carcinoma cells at the indicated number of days post-plating, measured by quantitative RT-PCR (qPCR). A parallel culture that received regular changes of media (indicated as ‘MC’), harvested at 11 d, is shown for comparison. Histograms show the mean of three technical replicates ± SEM of a representative experiment (experiments were repeated at least 3 times). Data is expressed as relative quantification (RQ) after normalization to *ACTB* and *CYPA*, and is calibrated to levels at day 2. Cell counts at the time of harvesting are provided ± SEM (black dotted line; average of three counts expressed as proportion of a confluent population). Right, comparison between replication domain (*CIZ1*-RD), anchor domain (*CIZ1*-AD) and *CIZ1*-F in MCF-7 cells at day 2 ± SEM, with no normalization to show relative levels. (E) As in (D) for MRC5 normal fetal lung fibroblast cells. Right, comparison between *CIZ1*-RD, *CIZ1*-AD and *CIZ1*-F at day 2. SEM for three technical replicates is shown. Primer and probe sequences are in Supplementary Table 2.

We next focused on CIZ1-F expression in cycling and quiescent cell populations, since CIZ1-F retains most regions involved in DNA-replication (Figure 1(b)). In detergent-treated cell populations, endogeneous CIZ1-F protein expression patterns changed on entry to quiescence, switching from nuclear localization in cycling cells, to nuclear exclusion with cytoplasmic signal at confluence, to nearly undetectable in quiescence (Figure 3(a-b)). Importantly, when quiescent cell populations were subjected to full NM-extraction, all signal was lost when cells were treated with DNase, demonstrating that quiescent cell populations do not retain any CIZ1-F on the NM (Figure 3(c)).

*CIZ1*-F transcript levels were also assessed in cycling, contact-inhibited and serum-deprived cells, and compared to amplicons in *CIZ1*-RD and *CIZ1*-AD (Figure 3(d-e)). Overall, *CIZ1*-F transcript is approximately 50-fold lower than ‘total’ *CIZ1* mRNA in cycling cells, which is broadly consistent with the frequency of expressed sequence tags in NCBI UniGene (accessed 03/03/2018), where 11 of 875 sequences (all from cancers) are *CIZ1*-F. *CIZ1*-RD and *CIZ1*-AD levels remain relatively stable during proliferative growth, and slightly increase during growth rate decline caused by serum starvation. However, *CIZ1*-F mRNA expression fell dramatically as proliferation rate dropped (Figure 3(d); Supplementary Figure 3(a)), and recovered again upon addition of fresh serum. In contrast, contact-inhibited normal fetal lung fibroblast MRC5 cells did not resume proliferation or restore *CIZ1*-F expression when boosted with serum (Figure 3(e)). This suggests that stable contact-induced growth arrest suppresses expression of *CIZ1*-F. In the same MCF-7 cell populations we observed additional effects on *CIZ1* alternative splicing, most notably increased *CIZ1*-S (Supplementary Figure 3(a)) an isoform reported to be upregulated in Alzheimer’s disease (Supplementary Table 1), and changes in *CIZ1*-Δ4 (Supplementary Figure 3(b)) [2,3]. Together the data show that CIZ1-F mRNA and protein are expressed in proliferating cells and inhibited by both serum-deprivation-induced proliferation arrest (MCF-7), and contact-inhibition-induced proliferation arrest (MRC5).

### *CIZ1*-F mRNA is expressed in G1 following mitosis (M-G1) but not G1 following quiescence (Q-G1)

**Figure 4.**
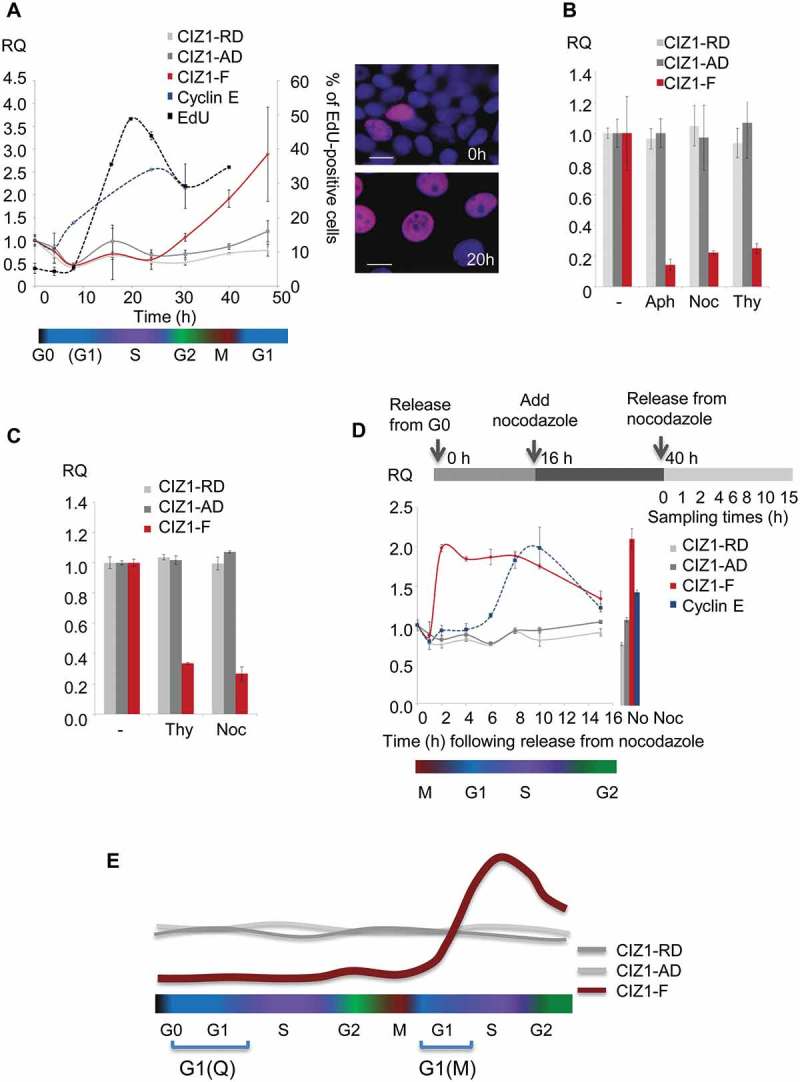
*CIZ1*-F transcript is expressed in G1-phase. (a) Quantitative RT-PCR showing *CIZ1*-F transcript (red) in MCF-7 cells during the first cell cycle following release from contact-inhibition and serum-deprivation induced cell cycle arrest, relative to *ACTB* and *CYPA* housekeeping genes, and calibrated to unreleased cells. *CIZ1* anchor domain (*CIZ1*-AD) and replication domain (*CIZ1*-RD) expression (gray lines), as well as cyclin E1 (blue) are shown for comparison. Data show means of four experiments ± SD. The percentage of cells that incorporated EdU into newly synthesized DNA during a 30 min pulse at the indicated times is shown for comparison (black line; two biological repeats ± SD). Note that error bars for cyclin E1 are very small (range, 0.00001–0.008). Cell cycle stages (estimates based on EdU-incorporation and cyclin-expression) are indicated below. Images on the right show EdU (purple) and total DNA (blue) before (0 h) and after (20 h) release. Bar is 10 microns. (b) *CIZ1*-AD, *CIZ1*-RD and *CIZ1*-F transcript in MCF-7 cells 31 h post-release from quiescence, in the presence and absence of aphidicolin (Aph), nocodazole (Noc) and thymidine (Thy), showing mean of three biological replicates ± SEM. (c) As in (b), but for MRC5 normal lung fetal fibroblast cells. Mean RQ of three technical replicates is expressed relative to cycling samples (± SEM). (d) As in (a), but showing release of MCF-7 cells from a 24 h nocodazole-arrest applied 16 h after release from quiescence. Synchronization strategy is indicated above the graph. Graph shows transcript levels (mean of three technical replicates ± SEM), at the indicated hours after release from arrest, with cell cycle stage (based on cyclin E expression) illustrated below. Right, control sample without nocodazole (40 h post-release from quiescence). (e) Schematic summarizing *CIZ1*-RD, *CIZ1*-AD and *CIZ1*-F mRNA expression levels during the first cell cycle following release from cell cycle arrest (Q-G1) and mitotic arrest (M-G1). Primer and probe sequences are in Supplementary Table 2.

To understand better the role of *CIZ1*-F in proliferation we analyzed its cell cycle expression profile. In order to generate synchronized populations, serum-deprived MCF-7 cells were released back into cycle in the presence of fresh serum and the nucleotide analog EdU (Figure 4(a)). The frequency of EdU-positive cells indicates that the population passes through S-phase between 10 and 20 h after exiting quiescence, peaking at 16 h (49% of nuclei; Figure 4(a)), and this is preceded as expected, by an increase in expression of cyclin E mRNA. However, *CIZ1*-F transcript does not begin to increase until after the majority of cells have passed through S-phase, recovering only at 31 h (Figure 4(a)). In contrast, *CIZ1* AD and RD expression remains relatively stable. When MCF-7 cells were exposed to the DNA synthesis inhibitors aphidicolin or thymidine, or arrested in mitosis by nocodazole upon release from quiescence, *CIZ1*-F expression was strongly suppressed compared to uninhibited cells (Figure 4(b)). Similar results were obtained with MRC5 cell populations treated with thymidine or nocodazole (Figure 4(c)). Thus *CIZ1*-F mRNA levels do not accumulate in either S-phase or mitosis and *CIZ1*-F is not expressed in Q-G1. However, when MCF7 cells were released from arrest with nocodazole in the M-phase following quiescence, *CIZ1*-F mRNA was expressed within 2 h, which is at least 4 h before cyclin E rises (Figure 4(d)). Thus, *CIZ1*-F mRNA is expressed in early G1 phase in the second cell cycle following release from quiescence, or M-G1 (Figure 4(e)).

### *CIZ1*-F mRNA expression in primary tumors and cancer cell lines

**Figure 5.**
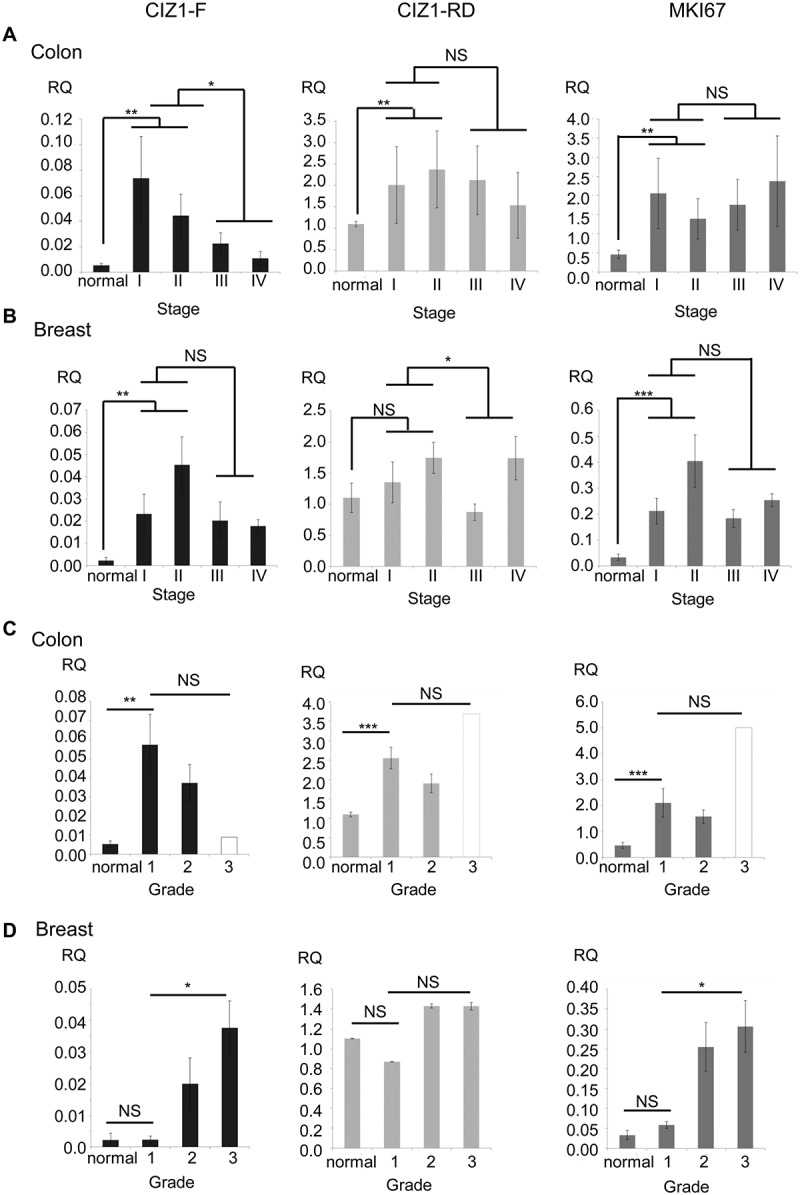
*CIZ1*-F is overexpressed in early stage human breast and colon cancer. (a) *CIZ1*-F and replication domain (*CIZ1*-RD) expression in 24 primary colon tumors and matched normal samples (Origene colon cancer cDNA array HCRT103), showing mean mRNA expression levels for the average of all matched normal tissues and the four cancer stage classifications. *CIZ1*-RD and *MKI67* are shown for comparison. Individual data per case can be found in Figure 4, and individual classifications and pathology notes accesses at www.origene.com. (b) As in (a), showing 5 normal samples and 43 primary breast cancer samples from the indicated stages (Origene breast cancer cDNA array BCRT102). Data for individual cases can be found in Figure 5. (c) As in (a) for the same 24 colon cancer samples and matched normal samples, showing mean mRNA expression grouped by grade. Grade 1: 50% late stage; grade 2: 38% late stage; grade 3: 100% late stage (1 case, white bar). Late stage refers to stage III and stage IV. (d) As in (b) for the same 5 normal samples and 43 primary breast cancer samples, showing mean mRNA expression grouped by grade. Grade 1: 100% late stage tumors; grade 2: 43% late stage tumors; grade 3: 27% late stage tumors. Late stage refers to stage III and stage IV. Mean RQ’s to the mean *CIZ1*-RD-expression of normal samples ± SEM are shown. Significant differences are indicated (NS, not significant). Primer and probe sequences are in Supplementary Table 2.

To confirm that *CIZ1*-F is overrepresented in cancer tissue, and to extend our analysis of lung tumors [1], we analyzed *CIZ1* mRNA in colon and breast cancer. Almost without exception colon cancer samples expressed elevated *CIZ1*-F compared to matched normal control tissue (*P *< 0.001, Related-Samples Wilcoxon Signed Rank Test; Figure 5(a); see Supplementary Figure 4 for individual sample data), and surprisingly the highest levels were in early stage tumors (*P *= 0.017 for early versus late stage, Mann-Whitney U-test). In contrast *CIZ1*-RD does not increase as dramatically in early stages, nor diminish significantly at later stages (*P *= 0.44 for the same comparison, Mann-Whitney U-test; Figure 5(a)). Thus the data indicate upregulation of *CIZ1*-F specifically in early stage colon cancer. Similar results were obtained with breast tumors with higher *CIZ1*-F expression in tumor tissue compared to normal breast tissues (*P *= 0.016, Mann-Whitney U-test), whereas expression of *CIZ1*-RD was not significantly different (Figure 5(b); see Supplementary Figure 5 for individual data) [1]. Again *CIZ1*-F levels were most elevated in early stage tumors (*P *= 0.006 when compared to normal tissues, Mann-Whitney U-test). Thus the profile of *CIZ1*-F expression across tumor stage is similar for both breast cancer and colon cancer, and further preliminary analysis of lung and urinary bladder cancers returned similar trends (Supplementary Figure 6), suggesting that the stage profile may be common to other tumor types.

**Figure 6.**
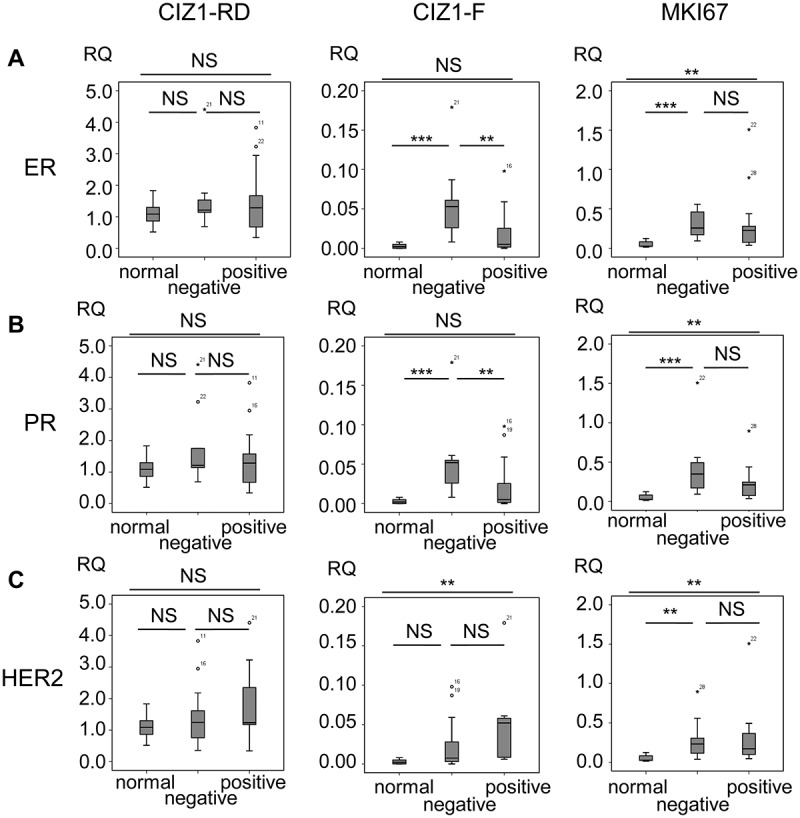
*CIZ1*-F expression is increased in ER-negative tumors. Box-plots showing *CIZ1* replication domain (*CIZ1*-RD; left), *CIZ1*-F (middle) and *MKI67* (right) expression in (a) normal samples and estrogen receptor (ER)-positive and -negative tumors, (b) normal samples and progesterone receptor (PR)-positive and -negative tumors, and (c) normal samples and HER-2-positive and -negative tumors. RQ’s are expressed relative to mean *CIZ1*-RD-expression of normal samples. Significant differences between subgroups are indicated (Mann Whitney U-tests; NS, not significant). Primer and probe sequences are in Supplementary Table 2.

When grouped by tumor grade rather than stage, the greatest elevation was at lowest grade for colon cancer (Figure 5(c)), but for breast cancer *CIZ1*-F levels increased with grade (*P *= 0.029, Kruskall-Wallis Test; Figure 5(d)). This is not surprising however since, in this series, most low-grade breast cancers were high-stage, and vice versa (see Figure 5(d)).

To evaluate the relationship with proliferation, the transcript level of the proliferation marker *MKI67* was measured in the same breast and colon samples (Figure 5). For colon, although *MKI67* and *CIZ1*-F levels correlated closely when the matched normal tissues were included in the analysis (*P *= 0.0020; Pearson Correlation), no correlation was found between levels across the cancer samples only, whereas *CIZ1*-RD was strongly correlated to *MKI67* levels by both analyzes (*P *≤ 0.00043; Pearson Correlation). Although less obvious for breast tumors, again no significant correlation across the full set for *CIZ1*-F and *MKI67* could be identified (*P *= 0.058; Pearson Correlation), compared to correlation with RD (*P *≤ 0.0050; Pearson Correlation). When performing linear regression with both *CIZ1*-RD and *CIZ1*-F in colon or breast samples, only *CIZ1*-RD significantly relates to *MKI67* expression in all analyzes (*P *≤ 0.0050). Thus, unlike *CIZ1*-RD, *CIZ1*-F does not mirror expression of *MKI67* and therefore does not seem to have a simple relationship with cell proliferation.

Interestingly, *CIZ1*-F levels correlated with hormone receptor status in breast tumors. ER and PR negative tumors have significantly higher *CIZ1*-F transcript levels than ER or progesterone receptor (PR) positive tumors (*P *= 0.005, Mann-Whitney U-test; Figure 6(a-b)), while HER2 status was not correlated (Figure 6(c)). This is not the case for *CIZ1*-RD, arguing for a relationship between CIZ1-F and ERα and PR in primary tumors.

## Discussion

Here we demonstrate that CIZ1-F is part of the RNA-dependent nuclear-matrix and elevated in early-stage cancers including hormone receptor negative, but not positive breast tumors. We show that CIZ1-F is distinct from other forms of CIZ1 and may have potential as a biomarker of early-stage disease.

### CIZ1-F is part of the RNA-dependent NM and distinct from full-length CIZ1

CIZ1 has multiple functions, both within and outside the context of DNA replication and the cell cycle, and its complexity is increased by the existence of multiple splice variants (Supplementary Table 1) [1–7,18]. We and others have shown that CIZ1 is a NM protein, defined by its resistance to extraction of chromatin [3,8], which suggests that its function is connected to nuclear architecture, possibly contributing to the spatial regulation of nuclear processes [6–8,18,24]. Although the NM appears to be corrupted in certain types of cancer [25] previous work [26–28] and our own experiments indicate that MCF-7 cells retain a NM structure to some extent. In MCF-7 cells both CIZ1-RD and CIZ1-F, as well as ERα, resist co-extraction with chromatin, indicating the presence of a non-chromatin stabilizing structure with which they are associated. This is of interest because CIZ1-F lacks the previously characterized NM anchor domain encoded by the C-terminal end of CIZ1. Moreover CIZ1-F is actually revealed by removal of chromatin, suggesting that the ARF-encoded epitope is normally in close proximity to chromatin, and masked by it. Further distinguishing CIZ1-F from full-length CIZ1 [8] is its behavior in quiescent cells. Here, CIZ1-F is excluded from the nucleus and not evident even after removal of chromatin, suggesting that its role is exclusively in cycling cells. Finally, though CIZ1-F is co-extracted with the RNA fraction of the NM (Figure 2(g)), it does not normally reside at the Xi implying that, unlike full-length CIZ1 this is not mediated by interaction with Xist lncRNA [9,10]. Future research should now focus on assigning a function to CIZ1-F. Since the unique junction sequence is a very limited target, we were not able to achieve specific CIZ1-F knockdown using an siRNA approach (data not shown).

### Biomarker potential for early stage cancers

Previous reports have shown overexpression of CIZ1 in colon [29,30], gallbladder [21], lung [31], and prostate cancer [32], as well as ependymomas, gliomas, and medulloblastomas [33], and it has also been reported that *CIZ1* knockout mice develop leukaemias and lymphomas [9,34]. Moreover, splice variants of CIZ1 appear to have lineage-specific functions [4] some of which are over-represented in human tumors; notably CIZ1-Δ4 in Ewings tumor and CIZ1-B in lung tumors [2,5]. However, for most published analyzes it is not clear which variants are reported on, and in some cases transcript detection tools that are reported to reflect *CIZ1* levels may actually reflect shifts in variant expression. Here we use validated and specific tools to demonstrate that *CIZ1*-F is upregulated in tumors of the colon and breast, that upregulation is most pronounced in early stage (I and II) disease, and is not directly correlated with proliferation. We also note that the association of *CIZ1*-F with tumor grade varies between tumor types, so although disrupted nuclear architecture and altered NM is a common feature of poorly differentiated and aggressive cancers [25,35], we cannot at this stage draw a correlation with suppression of CIZ1-F. In addition, our analyzes have currently been limited to *CIZ1*-F mRNA levels. Future studies should determine CIZ1-F protein expression patterns in primary tumor samples. Perhaps most useful is the clear correlation with hormone receptor status of breast tumors. Previous analysis has identified *CIZ1* as an estrogen-responsive gene with estrogen-response elements in its promoter [12]. Moreover, the N-terminal domain of CIZ1 protein can also interact with ER, conferring hypersensitivity to estrogen in animal models and enhancing the tumourigenicity of breast cancer cells [12]. Though alternative splicing was not addressed in this study, a contribution of CIZ1-F to the cells response to estrogen is likely because the ER interaction domain is partially retained in CIZ1-F, and it has two additional LXXLL nuclear receptor binding motifs [22] encoded by its unique C-terminal ARF. Contrary to published results [12] we did not observe induction of *CIZ1* upon stimulation with estrogen for 24 h. A possible explanation for the apparent discrepancy is that different *CIZ1* primers were used previously [12], which may well have reported on alternative splicing of *CIZ1* exon 8 rather than overall levels. In our study we detected *CIZ1*-RD and *CIZ1*-AD amplicons in regions unrelated to exon 8 in order to avoid this highly variable region of *CIZ1*. CIZ1 binding to (PR) has not been previously studied; though it has been shown that, in the context of NCoA-1/SRC-1, PR also requires two LXXLL domains [36]. Thus, while we could find no functional response to estrogen in MCF-7 cells, the presence of these domains and the correlation with breast tumor receptor status supports a relationship *in vivo*.

### Different CIZ1 splice variants at different cell proliferation stages

Transcript expression profile also distinguishes *CIZ1*-F from other variants of *CIZ1*. *CIZ1*-F has a distinct cell cycle-specific profile with low levels in G0, and delayed upregulation beyond the first cell cycle, which is at variance with the relatively constant expression of *CIZ1*-RD and *CIZ1*-AD amplicons (Figure 4(e)). In these studies we also noted proliferation state dependent changes in the *CIZ1*-S and *CIZ1*-Δ4 variants. In fact in MCF-7 cells, they seem to be inversely related to *CIZ1*-F, with *CIZ1*-S and *CIZ1*-Δ4 being more highly expressed in arrested cells (Supplementary Figure 3). It is worth noting that both *CIZ1*-F and *CIZ1*-S variants are the product of exon 8 splicing events, and that exon 8 splicing patterns have been implicated in Alzheimer’s disease and cervical dystonia [3,14].

In conclusion, this exploratory study has convincingly distinguished CIZ1-F from CIZ1, revealed association with the RNA-dependent NM, and highlighted an unusual cell cycle expression pattern. It also supports a relationship with estrogen responsive cancers and suggests that CIZ1-F may have potential as a biomarker.

## Materials & methods

### Cell culture and tumor samples

MCF-7 breast cancer cells were cultured in minimal essential medium (cat. 21090–022; Gibco, Life Technologies, Thermo Fisher Scientific [TFS] Inc) in the presence of 10% fetal calf serum (FCS; cat. FB-5815 Biosera, Boussens, France) and 1X penicillin-streptomycin-glutamine (PSG; cat. 10378–016; Gibco, Life Technologies, TFS Inc). MRC5 normal lung fetal fibroblast cells were grown in Dulbecco’s Modified Eagle Medium (cat. 21885–025; Gibco, Life Technologies, TFS Inc) in the presence of 10% FCS and 1X PSG. MCF-10A normal breast cells were cultured as previously described [37]. The cell lines were tested and authenticated in August 2017 using PCR-single-locus-technology (Eurofins Medigenomix Forensik GmbH). TissueScan^TM^ Cancer Survey cDNA array (CSRT103; Origene) was used to assess *CIZ1*-F levels in different human cancer types. The breast cancer cDNA array BCRT102 (Origene) was used to screen 43 breast cancer samples and 5 normal breast tissues. The colon cancer cDNA array HCRT103 (Origene) was used to screen 24 colon cancer samples with matched normal tissues.

### Quantitative real-time PCR (qRT-PCR)

RNA was isolated using Trizol® Reagent (cat. 15596–026; Ambion RNA, Life Technologies, TFS Inc) and reverse transcribed into cDNA using Superscript® III First-Strand Synthesis System (cat. 18080–051; Life Technologies, TFS Inc). Quantitative RT-PCR was performed using SYBR green (FastSYBR® Green Mastermix, cat. 4385612; Applied Biosystems® [AB], TFS Inc) or Taqman (Taqman® Fast Universal PCR Master Mix, cat. 4352042; AB, TFS Inc) reagents using the AB StepOnePlus^TM^ Real-Time PCR Systems (TFS Inc). The following program was used: 95°C for 20 s, followed by 40 cycles of 95°C for 3 s and 60°C for 30 s. Data was analyzed using the StepOne Software v. 2.3 (AB, TFS Inc). Origene® cancer cDNA array plates were processed with the AB 7300 system (TFS Inc) using the TaqMan® Universal Master Mix II (cat. 4440040; AB, TFS Inc) using the following program: 50°C for 2 min, 95°C for 10 min, followed by 50 cycles of 95°C for 15 s and 60°C for 1 min.

Primers were designed using Primer 3 Plus (http://primer3plus.com/), except for the *ACTB* primers [5] and *CYPA* primers [38], which were previously described, and cyclin D1 and cyclin E1 primers, which were retrieved from qPrimerDepot (http://primerdepot.nci.nhi.gov). All primers are listed in Supplementary Table 2. *CIZ1*-RD was detected with primers in exon 6/7 and *CIZ1*-AD with primers in exon 15/16 (Figure 1(a), Supplementary Table 2). For the Origene cDNA array plates, *CIZ1*-F was detected with primers 23–25, *CIZ1*-RD with primers 20–22 and *MKI67* using a Taqman Assay (assay no. Hs01032443_m1). All expression levels have been normalized to the mean expression of *ACTB* and *CYPA* unless stated differently.

### Constructs and transfection

Human *CIZ1*-F was cloned from cDNA from MCF-7 cells using PCR with DreamTaq (cat. EP1701; TFS Inc) and T/A cloning into the pGEM®-T Easy Vector System I (cat. A1360; Promega) and verified by sequencing. *CIZ1*-F was subsequently transferred using restriction enzymes (PmlI and SanDI, cat. ER0361 and FD2164, respectively; TFS Inc) into the pEGFP-C3 vector, allowing direct comparison with full-length *CIZ1*, containing all translated exons (2–17), also cloned into pEGFP-C3 as part of a previous study [5]. Both plasmids were transiently transfected into MCF-7 cells using the *Trans*IT®-3T3 and *Trans*IT®-LT1 Transfection Kits (cat. MIR 2180 and MIR 2300, respectively; Mirus Bio LLC).

### Antibodies

CIZ1-F specific antibody was made by immunizing rabbits with two peptides (Figure 1(c)) from the unique CIZ1-F ARF-encoded peptide (Covalab UK Ltd). Antisera from two rabbits were pooled and immunopurified against peptide 2 (which had no significant similarity to any other protein) and used for Western Blot and immunofluorescence (dilutions 1:100–1:500 and 1:50, respectively). CIZ1-F blocking peptides were used in a 1:500 dilution. Mouse monoclonal antibody (hC221a) against CIZ1 was raised by Fusion Antibodies against a recombinant human CIZ1 C-terminal fragment encoded by amino acids 678–898 in reference sequence UniProt Q9ULV3. In addition, purified CIZ1 1794 polyclonal antiserum [6] was used to detect CIZ1 without discriminating CIZ1-F. For immunofluorescence goat polyclonal anti-mouse-AlexaFluor488 conjugate or goat polyclonal anti-rabbit-AlexaFluor568 conjugate (cat. A11001 and A11011, respectively, Life Technologies, TFS) were used as appropriate. For Western Blot CIZ1 exon 5 antibody (cat. HPA-020380; Sigma-Aldrich) was used to detect CIZ1 without discriminating CIZ1-F. Actin (cat. A3853, Sigma Aldrich) was used as a loading control.

### Immunodetection

Protein extracts were obtained by washing cells with cold PBS and harvesting directly into SDS sample buffer (100 mM DTT, 2% SDS, 60 mM Tris pH 6.8, 0.001% bromophenol blue) at 95°C in the presence of 2 mM PMSF. Western blot was performed as previously described [6]. Bands were visualized using EZ-ECL (cat. 20-500-120; Biological Industries Ltd).

For immunofluorescence, cells grown on glass coverslips were transferred into PBS, washed with cytoskeletal (CSK) buffer (containing EDTA-free protease inhibitor tablets [cat. 05056489001; F Hoffmann-La Roche Ltd, Sigma Aldrich] and 1 mM DTT) with or without 0.1% Triton X-100 [39] for 30 s, then transferred to PBS and fixed for 20 min in 4% paraformaldehyde in PBS. Primary antibodies were incubated for 1.5 h at 37°C and secondary antibodies for 1 h at 37°C in the dark in antibody buffer (PBS, 10 mg/ml BSA, 0.2% SDS, 0.1% Triton X-100). Nuclei were counterstained with DAPI (VECTASHIELD Antifade Mounting Medium with DAPI, cat. H-1200; Vector Laboratories).

Fluorescent image data was collected with a Zeiss Axiovert microscope (Carl Zeiss) with a 63/1.40 oil immersion objective and an AxioCam camera (Carl Zeiss Vision) with Openlab software (version 4.0.2, Improvision). Images were processed using Adobe PhotoShop to reduce background to black and to overlay multicolor images. Where quantitative data is derived from the images, all capture parameters and image processing was identical for samples and controls within an experiment.

### Nuclear matrix extractions

Cells were fractionated as previously described [39,40]. In brief, coverslips were washed with detergent (0.1% Triton-X-100 in CSK), followed by the same buffer with 0.5 M NaCl. Cells were then incubated at 37°C for 1 h in DNase Buffer with or without DNase I (0.5 U/µl, cat. 000000004716728001; F Hoffmann-La Roche Ltd, Sigma Aldrich), RNase (cat. 000000011579681001; F Hoffmann-La Roche Ltd, Sigma Aldrich) or both nucleases in combination, then fixed for 20 min with 8% paraformaldehyde. Vanadyl Ribonucleoside Complex (cat. S1402S; New England Biolabs) was added at 1:80 to all buffers with the exception of the RNase containing treatment step and its preceding wash steps. Coverslips were counterstained with Hoechst33258 (1:100,000, cat. H3569; TFS) to verify removal of chromatin, and immunostained as indicated.

### Cell cycle synchronization

MCF-7 cells were synchronized by release from quiescence as described [41]. Cells were grown to confluence, then the medium was changed, and cells cultured for a further four days without further media changes and then released in serum-containing medium by 1:4 splitting. Alternatively, cells were incubated in the presence of nocodazole (0.04 µg/ml), aphidicolin (5 µg/ml) or thymidine (2.5 mM) for 24 h, and released from arrest by washing twice in PBS followed by addition of fresh medium. In order to verify synchrony, incorporation of EdU into newly synthesized DNA was measured using Click-iT® EdU Alexa Fluor® 555 (cat. C10338; Invitrogen, Life Technologies, TFS Inc).

### Statistical analysis

Statistical analysis was performed using IBM SPSS Statistics for Windows (version 21.0.0.0). Associations between expression levels and clinical parameters were calculated using the Mann Whitney U-test or the Kruskall-Wallis test, and for related samples using the Related-Samples Wilcoxon Signed Rank Test, as indicated. Relationships between expression levels amongst different genes were determined using Pearson Correlation and linear regression. All statistical tests were two-sided and all *P*-values were considered significant when < 0.05. Significance levels were indicated as follows: *, *P *< 0.05; **, *P *< 0.01; ***, *P *< 0.001.

## Abbreviations:

AB: Applied Biosystems
AD: anchor domain
ARF: alternative reading frame
CIZ1: CDKN1A-interacting zinc finger protein
CSK: cytoskeletal
ERα: estrogen receptor alpha
FCS: fetal calf serum
NM: nuclear matrix
PR: progesterone receptor
PSG: penicillin-streptomycin-glutamine
RD: replication domain
RQ: relative quantity
SD: standard deviation
SEM: standard error of mean
qRT-PCR: quantitative real-time PCR
TFS: Thermo Fisher Scientific
Xi: inactive X chromosome
Xist lncRNA: X-inactive specific transcript long non-coding RNA
