# Supplementary material for: CIZ1-F, an alternatively spliced variant of the DNA replication protein CIZ1 with distinct expression and localisation, is overrepresented in early stage common solid tumours
Source: Cell Cycle. 2018 Oct 6;17(18):2268–83. doi: 10.1080/15384101.2018.1526600 (PMC6226236; doi:10.1080/15384101.2018.1526600)
Supplement: Supplemental Material [file kccy-17-18-1526600-s001.zip › 1526600/Supplementary Figure 1.pptx]

## Slide 1
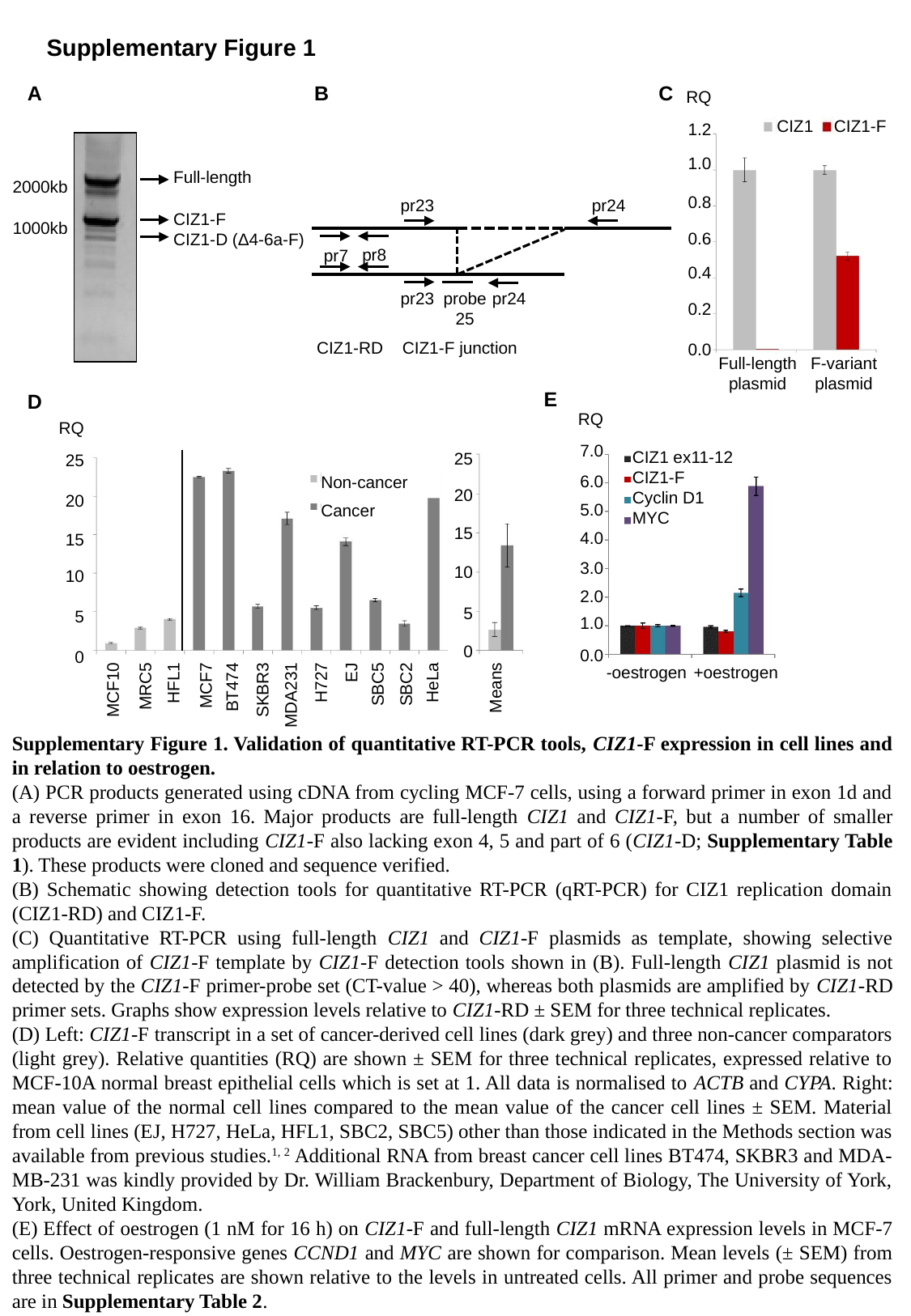

Supplementary Figure 1
A
B
C
RQ
1.2
1.0
0.8
0.6
0.4
0.2
0.0
Full-length plasmid
F-variant plasmid
CIZ1
CIZ1-F
Full-length
CIZ1-F
CIZ1-D (Δ4-6a-F)
2000kb
1000kb
pr23
pr24
pr8
pr7
pr23
probe 25
pr24
CIZ1-RD
CIZ1-F junction
E
D
RQ
7.0
6.0
5.0
4.0
3.0
2.0
1.0
0.0
-oestrogen
+oestrogen
CIZ1 ex11-12
CIZ1-F
Cyclin D1
MYC
RQ
25
20
15
10
5
0
25
20
15
10
5
0
Non-cancer
Cancer
MCF10
MRC5
HFL1
MCF7
BT474
SKBR3
MDA231
H727
EJ
SBC5
SBC2
HeLa
Means
Supplementary Figure 1. Validation of quantitative RT-PCR tools, CIZ1-F expression in cell lines and in relation to oestrogen.
(A) PCR products generated using cDNA from cycling MCF-7 cells, using a forward primer in exon 1d and a reverse primer in exon 16. Major products are full-length CIZ1 and CIZ1-F, but a number of smaller products are evident including CIZ1-F also lacking exon 4, 5 and part of 6 (CIZ1-D; Supplementary Table 1). These products were cloned and sequence verified.
(B) Schematic showing detection tools for quantitative RT-PCR (qRT-PCR) for CIZ1 replication domain (CIZ1-RD) and CIZ1-F.
(C) Quantitative RT-PCR using full-length CIZ1 and CIZ1-F plasmids as template, showing selective amplification of CIZ1-F template by CIZ1-F detection tools shown in (B). Full-length CIZ1 plasmid is not detected by the CIZ1-F primer-probe set (CT-value > 40), whereas both plasmids are amplified by CIZ1-RD primer sets. Graphs show expression levels relative to CIZ1-RD ± SEM for three technical replicates.
(D) Left: CIZ1-F transcript in a set of cancer-derived cell lines (dark grey) and three non-cancer comparators (light grey). Relative quantities (RQ) are shown ± SEM for three technical replicates, expressed relative to MCF-10A normal breast epithelial cells which is set at 1. All data is normalised to ACTB and CYPA. Right: mean value of the normal cell lines compared to the mean value of the cancer cell lines ± SEM. Material from cell lines (EJ, H727, HeLa, HFL1, SBC2, SBC5) other than those indicated in the Methods section was available from previous studies.1, 2 Additional RNA from breast cancer cell lines BT474, SKBR3 and MDA-MB-231 was kindly provided by Dr. William Brackenbury, Department of Biology, The University of York, York, United Kingdom.
(E) Effect of oestrogen (1 nM for 16 h) on CIZ1-F and full-length CIZ1 mRNA expression levels in MCF-7 cells. Oestrogen-responsive genes CCND1 and MYC are shown for comparison. Mean levels (± SEM) from three technical replicates are shown relative to the levels in untreated cells. All primer and probe sequences are in Supplementary Table 2.
