# Supplementary material for: CIZ1-F, an alternatively spliced variant of the DNA replication protein CIZ1 with distinct expression and localisation, is overrepresented in early stage common solid tumours
Source: Cell Cycle. 2018 Oct 6;17(18):2268–83. doi: 10.1080/15384101.2018.1526600 (PMC6226236; doi:10.1080/15384101.2018.1526600)
Supplement: Supplemental Material [file kccy-17-18-1526600-s001.zip › 1526600/Supplementary Figure 2.pptx]

## Slide 1
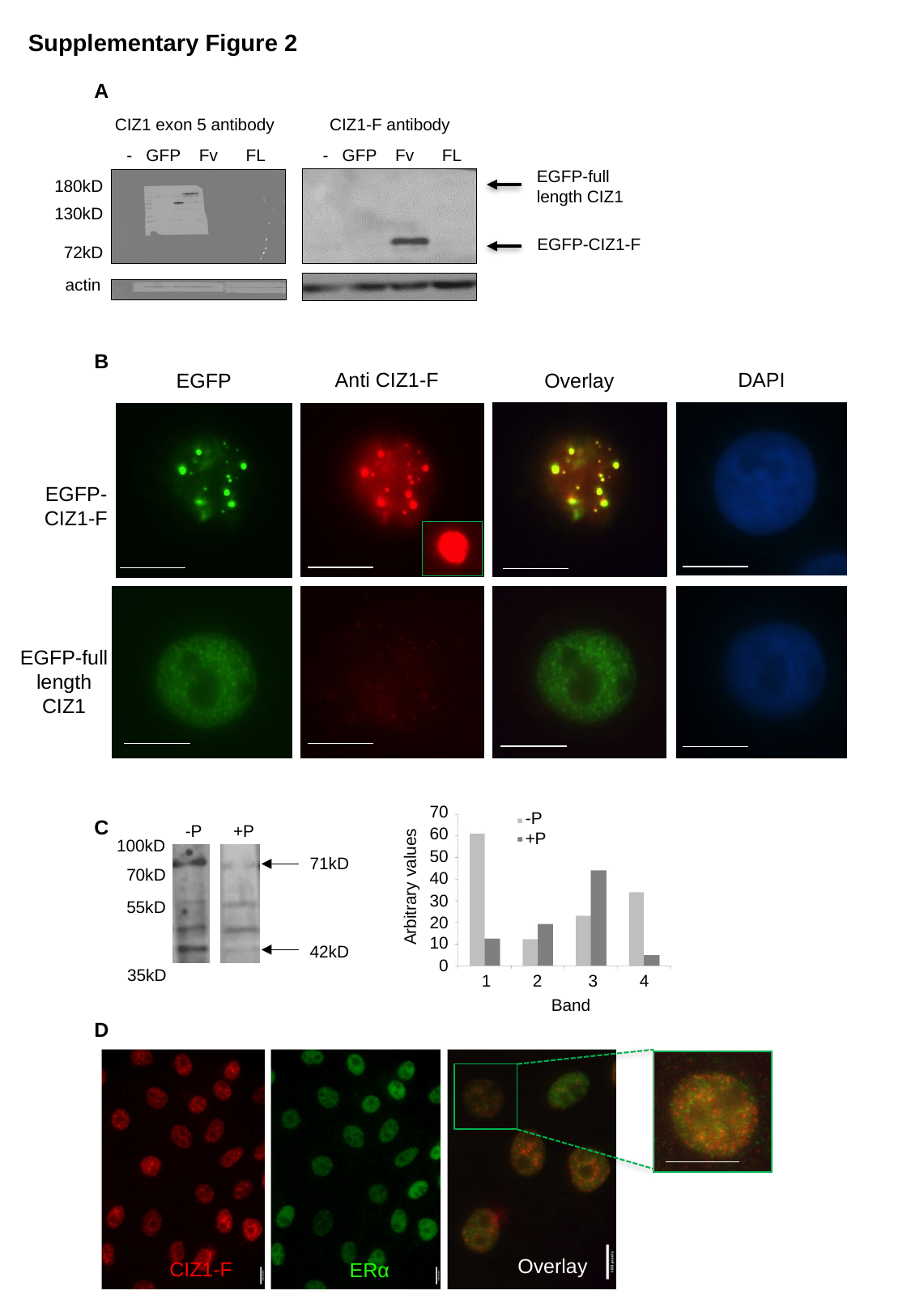

Supplementary Figure 2
A
CIZ1-F antibody
CIZ1 exon 5 antibody
actin
 - GFP Fv FL
- GFP Fv FL
180kD
130kD
72kD
EGFP-full length CIZ1
EGFP-CIZ1-F
B
DAPI
Anti CIZ1-F
EGFP
Overlay
EGFP-CIZ1-F
EGFP-full length CIZ1
70
60
50
40
30
20
10
0
Arbitrary values
-P
+P
1 2 3 4
Band
C
-P
+P
100kD
70kD
55kD
35kD
71kD
42kD
D
Overlay
CIZ1-F
ERα

## Slide 2
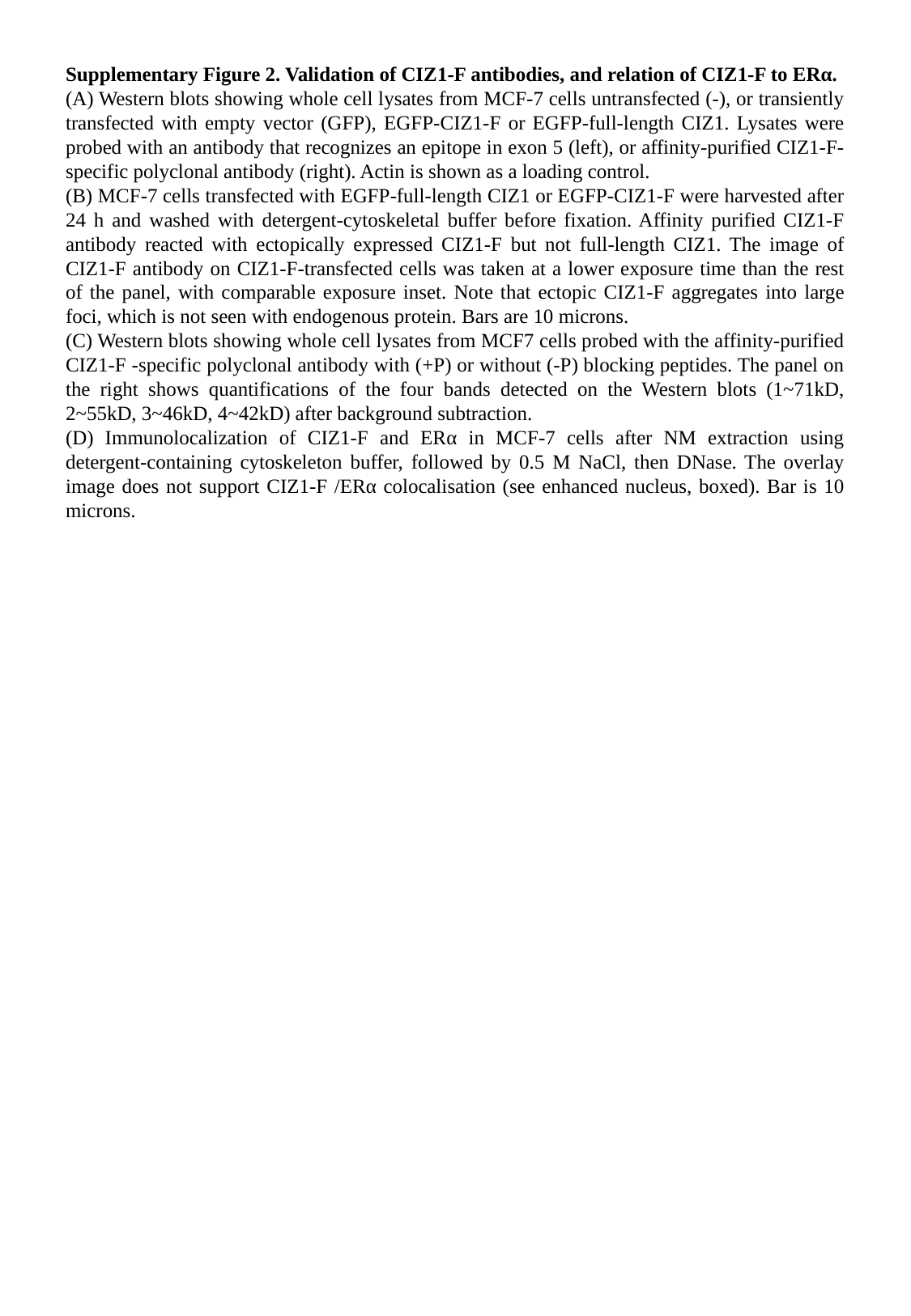

Supplementary Figure 2. Validation of CIZ1-F antibodies, and relation of CIZ1-F to ERα.
(A) Western blots showing whole cell lysates from MCF-7 cells untransfected (-), or transiently transfected with empty vector (GFP), EGFP-CIZ1-F or EGFP-full-length CIZ1. Lysates were probed with an antibody that recognizes an epitope in exon 5 (left), or affinity-purified CIZ1-F-specific polyclonal antibody (right). Actin is shown as a loading control.
(B) MCF-7 cells transfected with EGFP-full-length CIZ1 or EGFP-CIZ1-F were harvested after 24 h and washed with detergent-cytoskeletal buffer before fixation. Affinity purified CIZ1-F antibody reacted with ectopically expressed CIZ1-F but not full-length CIZ1. The image of CIZ1-F antibody on CIZ1-F-transfected cells was taken at a lower exposure time than the rest of the panel, with comparable exposure inset. Note that ectopic CIZ1-F aggregates into large foci, which is not seen with endogenous protein. Bars are 10 microns.
(C) Western blots showing whole cell lysates from MCF7 cells probed with the affinity-purified CIZ1-F -specific polyclonal antibody with (+P) or without (-P) blocking peptides. The panel on the right shows quantifications of the four bands detected on the Western blots (1~71kD, 2~55kD, 3~46kD, 4~42kD) after background subtraction.
(D) Immunolocalization of CIZ1-F and ERα in MCF-7 cells after NM extraction using detergent-containing cytoskeleton buffer, followed by 0.5 M NaCl, then DNase. The overlay image does not support CIZ1-F /ERα colocalisation (see enhanced nucleus, boxed). Bar is 10 microns.
