# Supplementary material for: CIZ1-F, an alternatively spliced variant of the DNA replication protein CIZ1 with distinct expression and localisation, is overrepresented in early stage common solid tumours
Source: Cell Cycle. 2018 Oct 6;17(18):2268–83. doi: 10.1080/15384101.2018.1526600 (PMC6226236; doi:10.1080/15384101.2018.1526600)
Supplement: Supplemental Material [file kccy-17-18-1526600-s001.zip › 1526600/Supplementary Figure 3.pptx]

## Slide 1
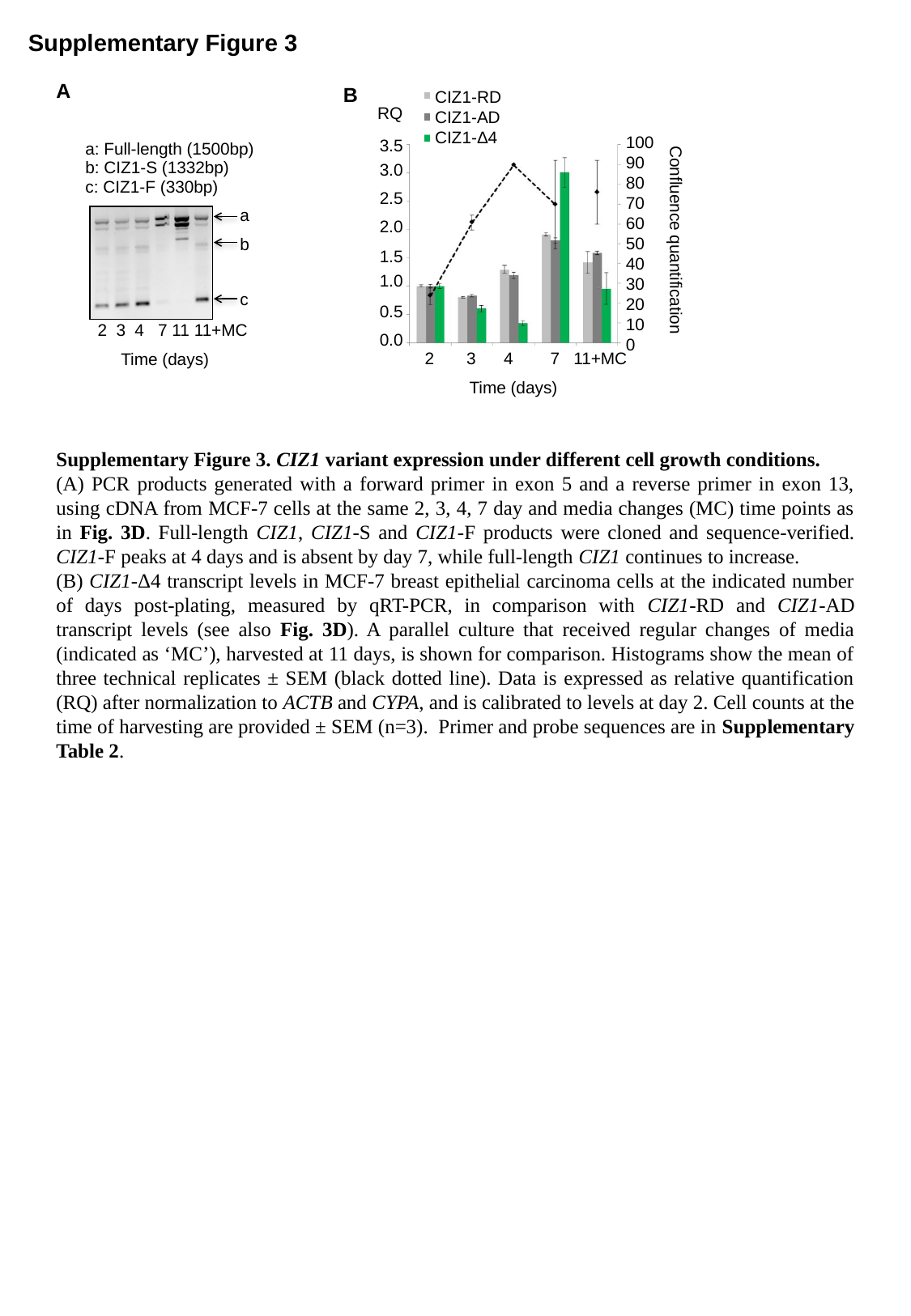

Supplementary Figure 3
A
B
CIZ1-RD
CIZ1-AD
CIZ1-Δ4
RQ
3.5
3.0
2.5
2.0
1.5
1.0
0.5
0.0
100
90
80
70
60
50
40
30
20
10
0
Confluence quantification
 2 3 4 7 11+MC
Time (days)
a: Full-length (1500bp)
b: CIZ1-S (1332bp)
c: CIZ1-F (330bp)
a
b
c
 2 3 4 7 11 11+MC
Time (days)
Supplementary Figure 3. CIZ1 variant expression under different cell growth conditions.
(A) PCR products generated with a forward primer in exon 5 and a reverse primer in exon 13, using cDNA from MCF-7 cells at the same 2, 3, 4, 7 day and media changes (MC) time points as in Fig. 3D. Full-length CIZ1, CIZ1-S and CIZ1-F products were cloned and sequence-verified. CIZ1-F peaks at 4 days and is absent by day 7, while full-length CIZ1 continues to increase.
(B) CIZ1-Δ4 transcript levels in MCF-7 breast epithelial carcinoma cells at the indicated number of days post-plating, measured by qRT-PCR, in comparison with CIZ1-RD and CIZ1-AD transcript levels (see also Fig. 3D). A parallel culture that received regular changes of media (indicated as ‘MC’), harvested at 11 days, is shown for comparison. Histograms show the mean of three technical replicates ± SEM (black dotted line). Data is expressed as relative quantification (RQ) after normalization to ACTB and CYPA, and is calibrated to levels at day 2. Cell counts at the time of harvesting are provided ± SEM (n=3). Primer and probe sequences are in Supplementary Table 2.
