# Supplementary material for: CIZ1-F, an alternatively spliced variant of the DNA replication protein CIZ1 with distinct expression and localisation, is overrepresented in early stage common solid tumours
Source: Cell Cycle. 2018 Oct 6;17(18):2268–83. doi: 10.1080/15384101.2018.1526600 (PMC6226236; doi:10.1080/15384101.2018.1526600)
Supplement: Supplemental Material [file kccy-17-18-1526600-s001.zip › 1526600/Supplementary Figure 4.pptx]

## Slide 1
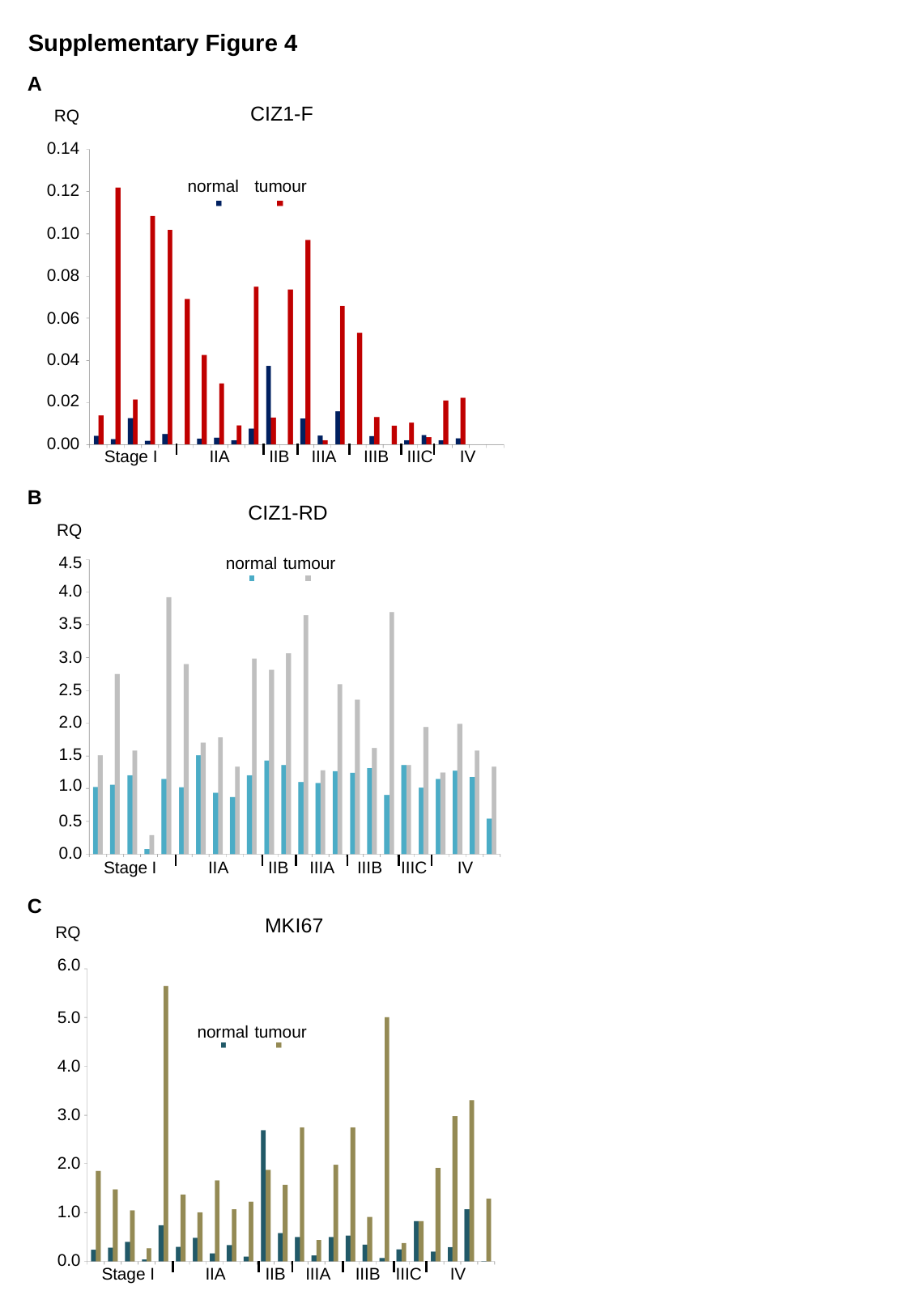

Supplementary Figure 4
A
CIZ1-F
RQ
0.14
0.12
0.10
0.08
0.06
0.04
0.02
0.00
Stage I
IIA
IIIA
IV
IIB
IIIB
IIIC
normal
tumour
RQ
B
CIZ1-RD
RQ
4.5
4.0
3.5
3.0
2.5
2.0
1.5
1.0
0.5
0.0
Stage I
IIA
IIIA
IIIC
IV
IIB
IIIB
tumour
normal
C
MKI67
RQ
6.0
5.0
4.0
3.0
2.0
1.0
0.0
IIB
IIIB
IIIC
IV
Stage I
IIA
IIIA
normal
tumour

## Slide 2
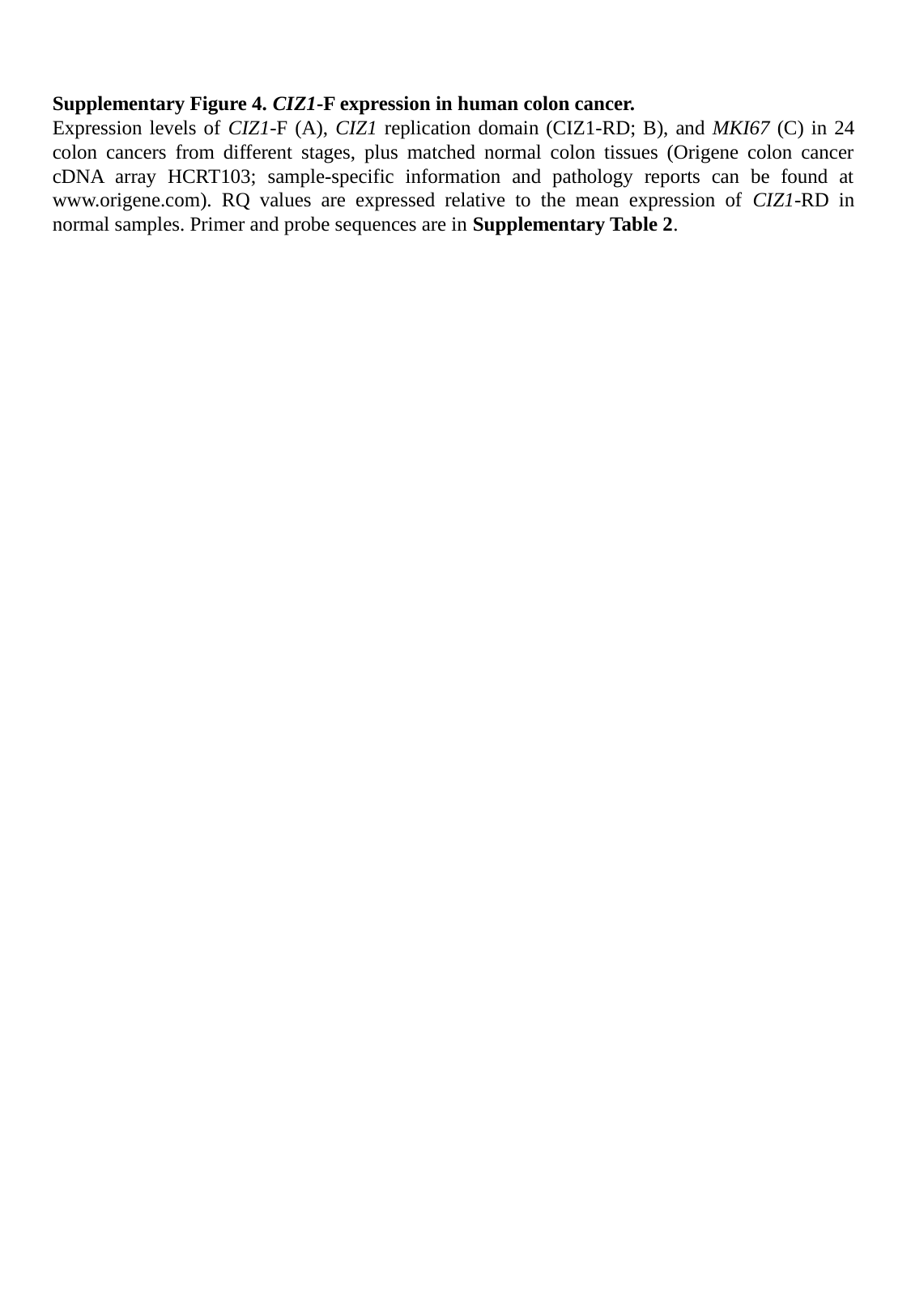

Supplementary Figure 4. CIZ1-F expression in human colon cancer.
Expression levels of CIZ1-F (A), CIZ1 replication domain (CIZ1-RD; B), and MKI67 (C) in 24 colon cancers from different stages, plus matched normal colon tissues (Origene colon cancer cDNA array HCRT103; sample-specific information and pathology reports can be found at www.origene.com). RQ values are expressed relative to the mean expression of CIZ1-RD in normal samples. Primer and probe sequences are in Supplementary Table 2.
