# Supplementary material for: CIZ1-F, an alternatively spliced variant of the DNA replication protein CIZ1 with distinct expression and localisation, is overrepresented in early stage common solid tumours
Source: Cell Cycle. 2018 Oct 6;17(18):2268–83. doi: 10.1080/15384101.2018.1526600 (PMC6226236; doi:10.1080/15384101.2018.1526600)
Supplement: Supplemental Material [file kccy-17-18-1526600-s001.zip › 1526600/Supplementary Figure 5.pptx]

## Slide 1
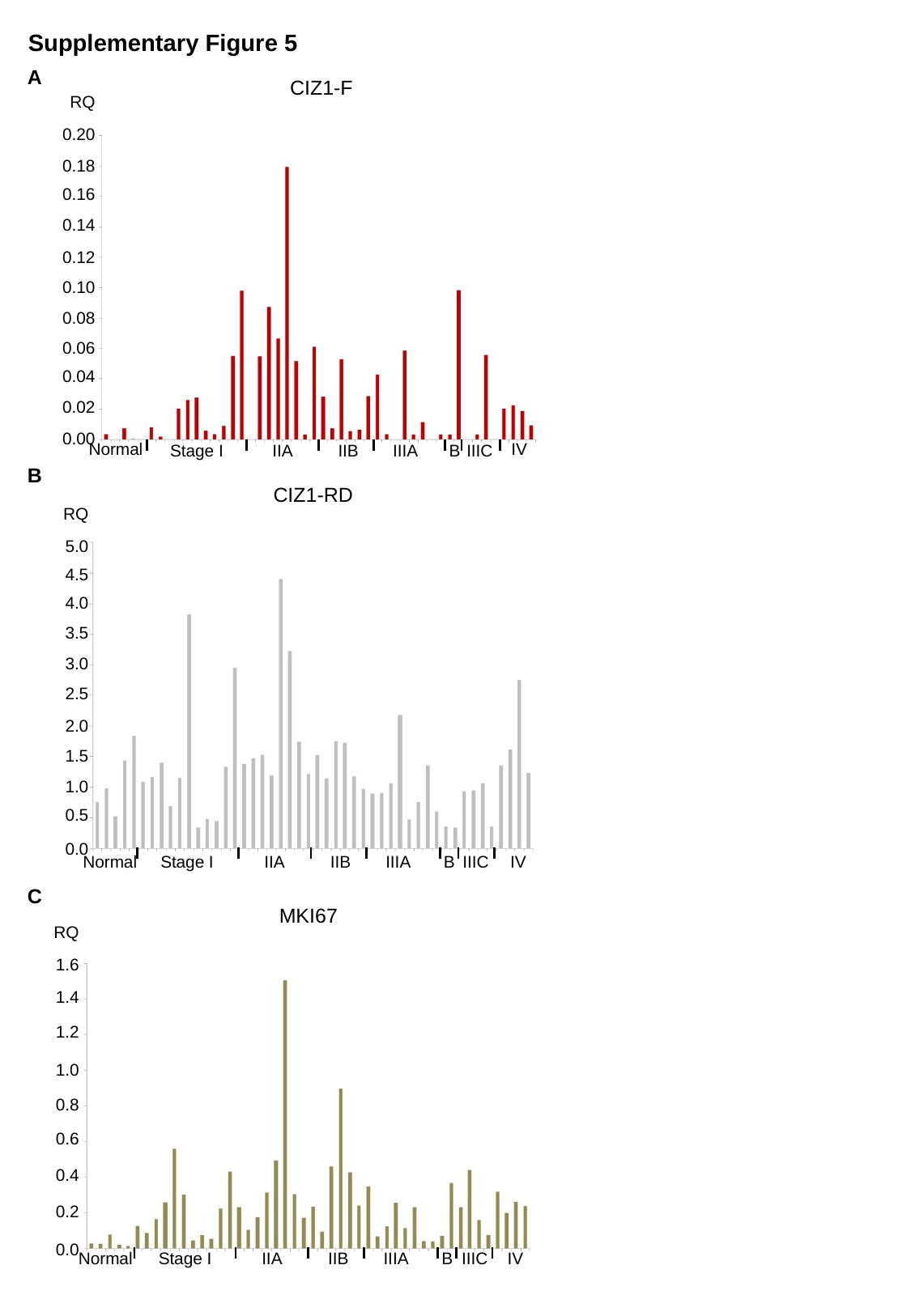

Supplementary Figure 5
A
CIZ1-F
RQ
0.20
0.18
0.16
0.14
0.12
0.10
0.08
0.06
0.04
0.02
0.00
Normal
IV
Stage I
IIA
IIB
IIIA
B
IIIC
B
CIZ1-RD
RQ
5.0
4.5
4.0
3.5
3.0
2.5
2.0
1.5
1.0
0.5
0.0
Normal
Stage I
IIA
IIB
IIIA
B
IIIC
IV
C
MKI67
RQ
1.6
1.4
1.2
1.0
0.8
0.6
0.4
0.2
0.0
Normal
IIIC
IV
Stage I
IIA
IIB
IIIA
B

## Slide 2
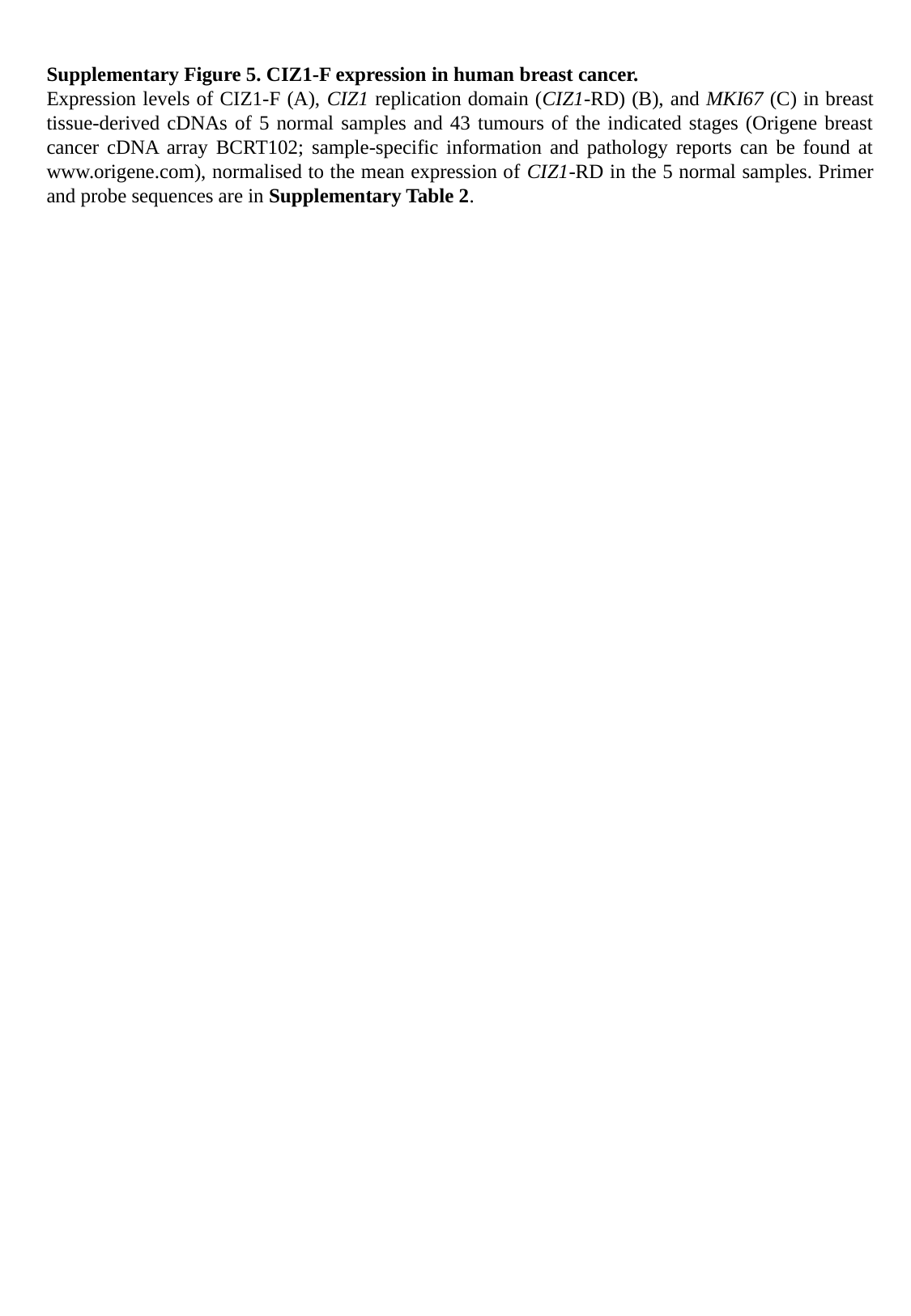

Supplementary Figure 5. CIZ1-F expression in human breast cancer.
Expression levels of CIZ1-F (A), CIZ1 replication domain (CIZ1-RD) (B), and MKI67 (C) in breast tissue-derived cDNAs of 5 normal samples and 43 tumours of the indicated stages (Origene breast cancer cDNA array BCRT102; sample-specific information and pathology reports can be found at www.origene.com), normalised to the mean expression of CIZ1-RD in the 5 normal samples. Primer and probe sequences are in Supplementary Table 2.
