# Supplementary material for: CIZ1-F, an alternatively spliced variant of the DNA replication protein CIZ1 with distinct expression and localisation, is overrepresented in early stage common solid tumours
Source: Cell Cycle. 2018 Oct 6;17(18):2268–83. doi: 10.1080/15384101.2018.1526600 (PMC6226236; doi:10.1080/15384101.2018.1526600)
Supplement: Supplemental Material [file kccy-17-18-1526600-s001.zip › 1526600/Supplementary Figure 6.pptx]

## Slide 1
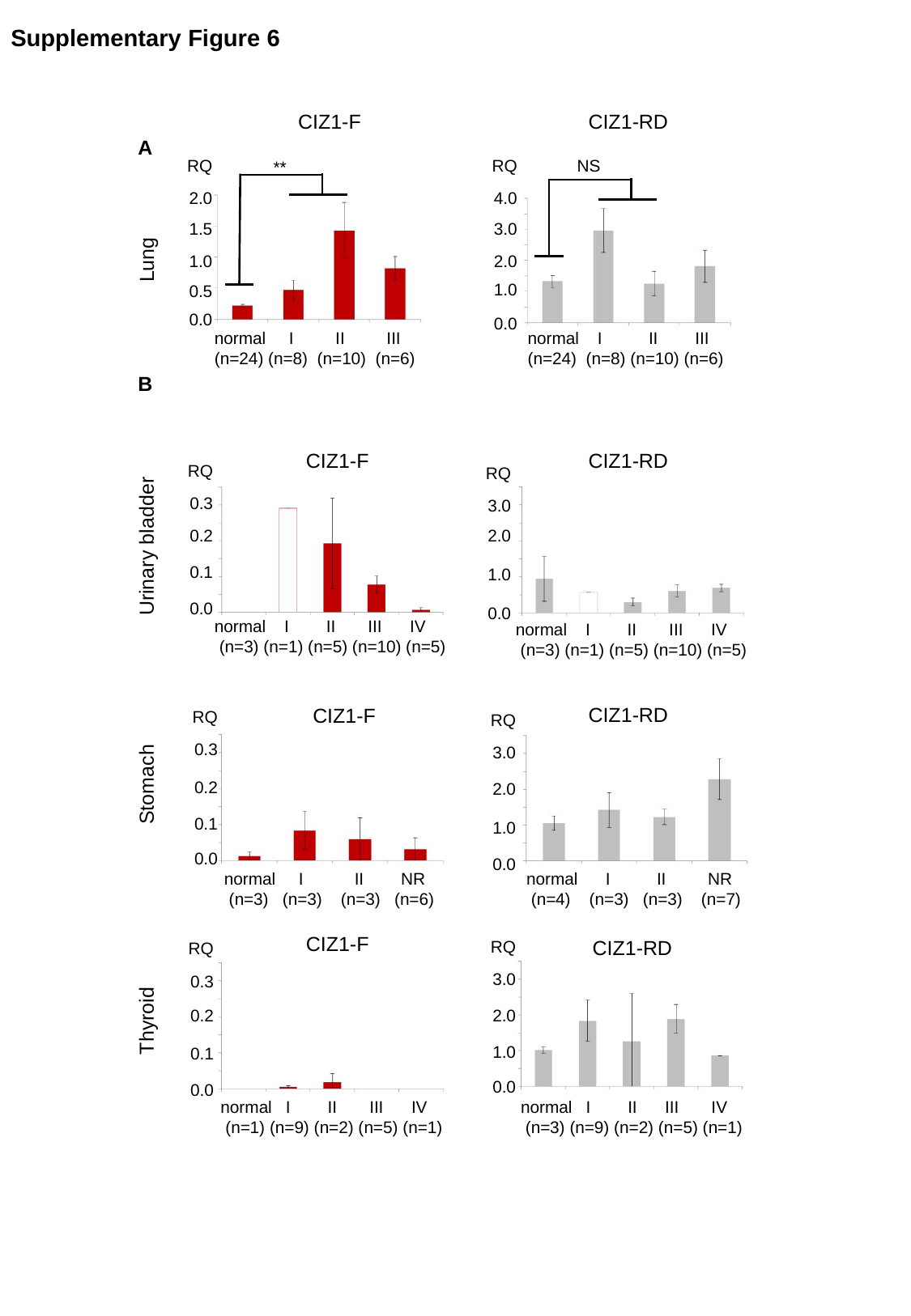

Supplementary Figure 6
CIZ1-F
CIZ1-RD
A
RQ
2.0
1.5
1.0
0.5
0.0
**
normal I II III
(n=24) (n=8) (n=10) (n=6)
RQ
4.0
3.0
2.0
1.0
0.0
NS
normal I II III
(n=24) (n=8) (n=10) (n=6)
Lung
B
CIZ1-F
CIZ1-RD
RQ
0.3
0.2
0.1
0.0
normal I II III IV
 (n=3) (n=1) (n=5) (n=10) (n=5)
RQ
3.0
2.0
1.0
0.0
 normal I II III IV
 (n=3) (n=1) (n=5) (n=10) (n=5)
Urinary bladder
RQ
0.3
0.2
0.1
0.0
normal I II NR
 (n=3) (n=3) (n=3) (n=6)
RQ
3.0
2.0
1.0
0.0
normal I II NR
 (n=4) (n=3) (n=3) (n=7)
CIZ1-RD
CIZ1-F
Stomach
CIZ1-F
CIZ1-RD
RQ
3.0
2.0
1.0
0.0
normal I II III IV
 (n=3) (n=9) (n=2) (n=5) (n=1)
RQ
0.3
0.2
0.1
0.0
normal I II III IV
 (n=1) (n=9) (n=2) (n=5) (n=1)
Thyroid

## Slide 2
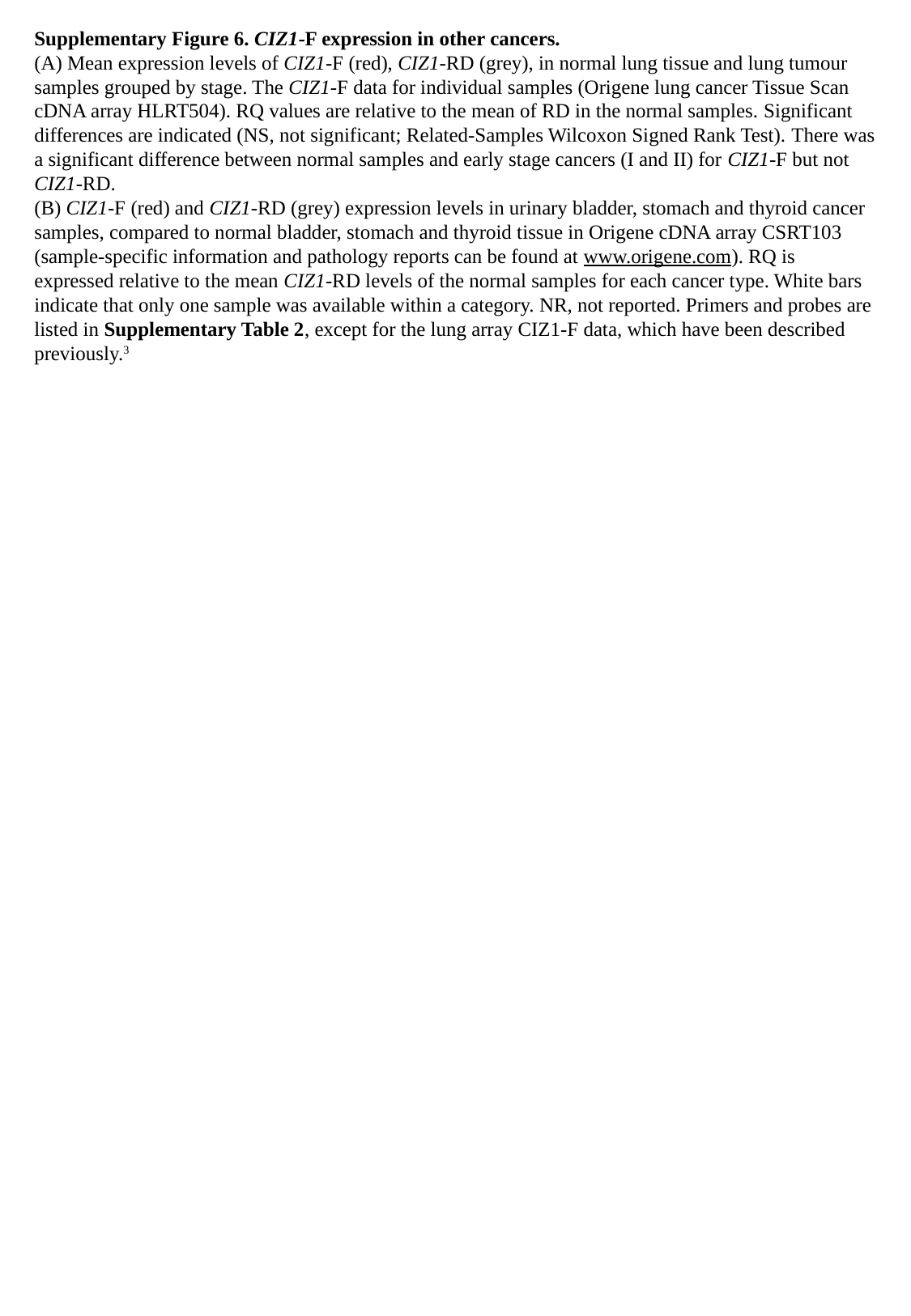

Supplementary Figure 6. CIZ1-F expression in other cancers.
(A) Mean expression levels of CIZ1-F (red), CIZ1-RD (grey), in normal lung tissue and lung tumour samples grouped by stage. The CIZ1-F data for individual samples (Origene lung cancer Tissue Scan cDNA array HLRT504). RQ values are relative to the mean of RD in the normal samples. Significant differences are indicated (NS, not significant; Related-Samples Wilcoxon Signed Rank Test). There was a significant difference between normal samples and early stage cancers (I and II) for CIZ1-F but not CIZ1-RD.
(B) CIZ1-F (red) and CIZ1-RD (grey) expression levels in urinary bladder, stomach and thyroid cancer samples, compared to normal bladder, stomach and thyroid tissue in Origene cDNA array CSRT103 (sample-specific information and pathology reports can be found at www.origene.com). RQ is expressed relative to the mean CIZ1-RD levels of the normal samples for each cancer type. White bars indicate that only one sample was available within a category. NR, not reported. Primers and probes are listed in Supplementary Table 2, except for the lung array CIZ1-F data, which have been described previously.3
