# Supplementary material for: CIZ1-F, an alternatively spliced variant of the DNA replication protein CIZ1 with distinct expression and localisation, is overrepresented in early stage common solid tumours
Source: Cell Cycle. 2018 Oct 6;17(18):2268–83. doi: 10.1080/15384101.2018.1526600 (PMC6226236; doi:10.1080/15384101.2018.1526600)
Supplement: Supplemental Material [file kccy-17-18-1526600-s001.zip › 1526600/Supplementary Figure Legends_RV.docx]

**Supplementary Figure Legends**

**Supplementary Figure 1. Validation of quantitative RT-PCR tools, *CIZ1*-F expression in cell lines and in relation to oestrogen.**

(A) PCR products generated using cDNA from cycling MCF-7 cells, using a forward primer in exon 1d and a reverse primer in exon 16. Major products are full-length *CIZ1* and *CIZ1*-F, but a number of smaller products are evident including *CIZ1*-F also lacking exon 4, 5 and part of 6 (*CIZ1*-D; **Supplementary Table 1**). These products were cloned and sequence verified.

(B) Schematic showing detection tools for quantitative RT-PCR (qRT-PCR) for CIZ1 replication domain (CIZ1-RD) and CIZ1-F.

(C) Quantitative RT-PCR using full-length *CIZ1* and *CIZ1*-F plasmids as template, showing selective amplification of *CIZ1*-F template by *CIZ1*-F detection tools shown in (B). Full-length *CIZ1* plasmid is not detected by the *CIZ1*-F primer-probe set (CT-value > 40), whereas both plasmids are amplified by *CIZ1*-RD primer sets. Graphs show expression levels relative to *CIZ1*-RD ± SEM for three technical replicates.

(D) Left: *CIZ1*-F transcript in a set of cancer-derived cell lines (dark grey) and three non-cancer comparators (light grey). Relative quantities (RQ) are shown ± SEM for three technical replicates, expressed relative to MCF-10A normal breast epithelial cells which is set at 1. All data is normalised to *ACTB* and *CYPA*. Right: mean value of the normal cell lines compared to the mean value of the cancer cell lines ± SEM. Material from cell lines (EJ, H727, HeLa, HFL1, SBC2, SBC5) other than those indicated in the Methods section was available from previous studies.^1, 2^ Additional RNA from breast cancer cell lines BT474, SKBR3 and MDA-MB-231 was kindly provided by Dr. William Brackenbury, Department of Biology, The University of York, York, United Kingdom.

(E) Effect of oestrogen (1 nM for 16 h) on *CIZ1*-F and full-length *CIZ1* mRNA expression levels in MCF-7 cells. Oestrogen-responsive genes *CCND1* and *MYC* are shown for comparison. Mean levels (± SEM) from three technical replicates are shown relative to the levels in untreated cells. All primer and probe sequences are in **Supplementary Table 2**.

**Supplementary Figure 2. Validation of CIZ1-F antibodies, and relation of CIZ1-F to ERα.**

(A) Western blots showing whole cell lysates from MCF-7 cells untransfected (-), or transiently transfected with empty vector (GFP), EGFP-CIZ1-F or EGFP-full-length CIZ1. Lysates were probed with an antibody that recognizes an epitope in exon 5 (left), or affinity-purified CIZ1-F-specific polyclonal antibody (right). Actin is shown as a loading control.

(B) MCF-7 cells transfected with EGFP-full-length CIZ1 or EGFP-CIZ1-F were harvested after 24 h and washed with detergent-cytoskeletal buffer before fixation. Affinity purified CIZ1-F antibody reacted with ectopically expressed CIZ1-F but not full-length CIZ1. The image of CIZ1-F antibody on CIZ1-F-transfected cells was taken at a lower exposure time than the rest of the panel, with comparable exposure inset. Note that ectopic CIZ1-F aggregates into large foci, which is not seen with endogenous protein. Bars are 10 microns.

(C) Western blots showing whole cell lysates from MCF7 cells probed with the affinity-purified CIZ1-F -specific polyclonal antibody with (+P) or without (-P) blocking peptides. The panel on the right shows quantifications of the four bands detected on the Western blots (1~71kD, 2~55kD, 3~46kD, 4~42kD) after background subtraction.

(D) Immunolocalization of CIZ1-F and ERα in MCF-7 cells after NM extraction using detergent-containing cytoskeleton buffer, followed by 0.5 M NaCl, then DNase. The overlay image does not support CIZ1-F /ERα colocalisation (see enhanced nucleus, boxed). Bar is 10 microns.

**Supplementary Figure 3. *CIZ1* variant expression under different cell growth conditions.**

(A) PCR products generated with a forward primer in exon 5 and a reverse primer in exon 13, using cDNA from MCF-7 cells at the same 2, 3, 4, 7 day and media changes (MC) time points as in **Fig. 3D**. Full-length *CIZ1*, *CIZ1*-S and *CIZ1*-F products were cloned and sequence-verified. *CIZ1*-F peaks at 4 days and is absent by day 7, while full-length *CIZ1* continues to increase.

(B) *CIZ1*-Δ4 transcript levels in MCF-7 breast epithelial carcinoma cells at the indicated number of days post-plating, measured by qRT-PCR, in comparison with *CIZ1*-RD and *CIZ1*-AD transcript levels (see also **Fig. 3D**). A parallel culture that received regular changes of media (indicated as ‘MC’), harvested at 11 days, is shown for comparison. Histograms show the mean of three technical replicates ± SEM (black dotted line). Data is expressed as relative quantification (RQ) after normalization to *ACTB* and *CYPA*, and is calibrated to levels at day 2. Cell counts at the time of harvesting are provided ± SEM (n=3). Primer and probe sequences are in **Supplementary Table 2**.

**Supplementary Figure 4. *CIZ1*-F expression in human colon cancer.**

Expression levels of *CIZ1*-F (A), *CIZ1* replication domain (CIZ1-RD; B), and *MKI67* (C) in 24 colon cancers from different stages, plus matched normal colon tissues (Origene colon cancer cDNA array HCRT103; sample-specific information and pathology reports can be found at www.origene.com). RQ values are expressed relative to the mean expression of *CIZ1*-RD in normal samples. Primer and probe sequences are in **Supplementary Table 2**.

**Supplementary Figure 5. CIZ1-F expression in human breast cancer.**

Expression levels of CIZ1-F (A), *CIZ1* replication domain (*CIZ1*-RD) (B), and *MKI67* (C) in breast tissue-derived cDNAs of 5 normal samples and 43 tumours of the indicated stages (Origene breast cancer cDNA array BCRT102; sample-specific information and pathology reports can be found at www.origene.com), normalised to the mean expression of *CIZ1*-RD in the 5 normal samples. Primer and probe sequences are in **Supplementary Table 2**.

**Supplementary Figure 6. *CIZ1*-F expression in other cancers.**

(A) Mean expression levels of *CIZ1*-F (red), *CIZ1*-RD (grey), in normal lung tissue and lung tumour samples grouped by stage. The *CIZ1*-F data for individual samples (Origene lung cancer Tissue Scan cDNA array HLRT504). RQ values are relative to the mean of RD in the normal samples. Significant differences are indicated (NS, not significant; Related-Samples Wilcoxon Signed Rank Test). There was a significant difference between normal samples and early stage cancers (I and II) for *CIZ1*-F but not *CIZ1*-RD.

(B) *CIZ1*-F (red) and *CIZ1*-RD (grey) expression levels in urinary bladder, stomach and thyroid cancer samples, compared to normal bladder, stomach and thyroid tissue in Origene cDNA array CSRT103 (sample-specific information and pathology reports can be found at [www.origene.com](http://www.origene.com)). RQ is expressed relative to the mean *CIZ1*-RD levels of the normal samples for each cancer type. White bars indicate that only one sample was available within a category. NR, not reported. Primers and probes are listed in **Supplementary Table 2**, except for the lung array CIZ1-F data, which have been described previously.^3^

**References**

1. Higgins G, Roper KM, Watson IJ, Blackhall FH, Rom WN, Pass HI, Ainscough JF, Coverley D. Variant Ciz1 is a circulating biomarker for early-stage lung cancer. Proc Natl Acad Sci U S A 2012; 109:E3128-35.

2. Rahman F, Ainscough JF, Copeland N, Coverley D. Cancer-associated missplicing of exon 4 influences the subnuclear distribution of the DNA replication factor CIZ1. Hum Mutat 2007; 28:993-1004.

3. Rahman FA, Aziz N, Coverley D. Differential detection of alternatively spliced variants of Ciz1 in normal and cancer cells using a custom exon-junction microarray. BMC Cancer 2010; 10:482.
