# Supplementary material for: CIZ1-F, an alternatively spliced variant of the DNA replication protein CIZ1 with distinct expression and localisation, is overrepresented in early stage common solid tumours
Source: Cell Cycle. 2018 Oct 6;17(18):2268–83. doi: 10.1080/15384101.2018.1526600 (PMC6226236; doi:10.1080/15384101.2018.1526600)
Supplement: Supplemental Material [file kccy-17-18-1526600-s001.zip › 1526600/Supplementary Tables.docx]

**Supplementary Tables**

| **Supplementary Table 1 - Overview of common CIZ1-variants*** | | | | | | | | | |
| --- | --- | --- | --- | --- | --- | --- | --- | --- | --- |
|  |  |  |  |  | |  |  | |  |
| **Name** | **Splicing event** | **Expression in normal tissue** | **Expression in disease** | **Cause** | | **Function** | **Subcellular expression pattern** | | **References** |
| ***Single events*** | | | | | | | | | |
| Ciz1-Δ3 | Deletion of exon 3 (fs; may be in combination with Δ4 and Δ6a) | In mouse testis during development | Unknown | May be developmentally regulated | | Unknown | | Unknown | Greaves^1^ |
| CIZ1-Δ4 (NP-94 in combination with Δ6a) | Deletion of exon 4 (in frame) | Expressed during mouse development and massively upregulated in differentiating male germ cells | Common in Ewing tumours | Expansion of upstream intronic splicing regulator | | Active in DNA replication | | Fails to form subnuclear foci and has a dominant-negative effect on other CIZ1 variants | Warder & Keherly^2^ Rahman,^3^ Greaves^1^ |
| CIZ1-Δ6a | Deletion of first five amino acids (DSSSQ) of exon 6 | Detected in human and mouse | Detected but not linked to disease | Unknown | | Unknown, possible phosphorylation site | | Unknown | Warder and Keherly,^2^ Greaves^1^ |
| CIZ1-F (f-variant) | Deletion of exon 9-11 and part of exon 8 and 12 (fs) | In several cell lines; downregulated in quiescence | Overexpressed in (early stage) tumours and cancer cell lines. Lower expression in ER-positive breast cancer | Unknown | | Associated with proliferation; involved in G_1_ | | Mainly nuclear in cycling cells, but cytoplasmic in contact-inhibited cells. Part of the RNA-component of the NM | Rahman^4^ and this study |
| CIZ1-M (m-variant) | Deletion of 84 bp from exon 8 (in frame) | Very low expression levels | Unknown, low | Unknown (repetitive sequences) | | Unknown | | Diffuse nuclear, abrogation of the association with the NM | Dahmcke^5^ |
| CIZ1-S (s-variant) | Deletion of 168 bp from exon 8 (in frame) | Lower in normal hippocampi | Upregulated in Alzheimer's disease | Unknown (repetitive sequences) | | Unknown | | Unknown | Dahmcke^5^ |
| CIZ1-B (b-variant) | Deletion of last 24 nt of exon 14 (in frame) | Low expression levels | Prevalent in lung cancer samples | Unknown | | Circulating biomarker | | Diffuse nuclear | Higgins^6^ |
| ***Combined events (selection)^+^*** | | | | | | | | | |
| CIZ1-Δ4-S (Δ4-s-variant) | Deletion of exon 4 and 168 bp from exon 8 (in frame) | Unknown | Unknown | Unknown | Unknown | | Unknown | | UniProt |
| CIZ1-D-F (d-f-variant) | Deletion of exon 4, 5 and first five amino acids (DSSSQ) of exon 6 and the CIZ1-F event (fs) | Unknown | Expressed in MCF-7 breast cancer cells | Unknown | Fs may lead to the use of an alternative TSS in exon 6 | | Unknown | | This study |
| CIZ1-D-S (d-s-variant) | Deletion of exon 4, 5 and DSSSQ and 168 bp from exon 8 (fs) | Unknown | Detected in MCF-7 breast cancer cells | Unknown | Fs may lead to the use of an alternative TSS in exon 6 | | Unknown | | Our unpublished data |
| CIZ1-S-Δ9 (s- Δ9-variant) | Deletion of 168 bp from exon 8, and exon 9 (fs) | Unknown | Ewing tumour cell lines and H727 lung cancer cell line | Unknown | Unknown | | Unknown | | Rahman^4^ |
| ECIZ1 (mouse) | Exclusion of exon 2 and part of exon 3, and exons 6 and 8 | Embryonic mouse tissues | Unknown | Unknown | Active in DNA replication | | Forms nuclear foci, but slightly less efficiently | | Coverley,^7^ Ainscough^8^ |
| *Note that additional *CIZ1* splicing events have been detected using an exon-junction microarray,^4^ which are not all listed here.  ^+^Many additional combinations have been identified. For instance, almost all *CIZ1* splicing events occur with or without Δ6a.  Abbreviations used: fs, frame-shift; NM, nuclear matrix; nt, nucleotides; TSS, translational start site. | | | | | | | | | |

| **Supplementary Table 2 - Primers and probes** | | |
| --- | --- | --- |
|  |  |  |
| **A) SYBR Green primers** | **Description** | **Sequence** |
| 1 | *ACTB* forward | 5' CAA CCG CGA GAA GAT GAC C 3' |
| 2 | *ACTB* reverse | 5' TCC AGG GCG ACG TAG CAC A 3' |
| 3 | *CCND1* forward | 5' TCC TCT CCA AAA TGC CAG AG 3' |
| 4 | *CCND1* reverse | 5' GGC GGA TTG GAA ATG AAC TT 3' |
| 5 | *CCNE1* forward | 5' GAA ATG GCC AAA ATC GAC AG 3' |
| 6 | *CCNE1* reverse | 5' TCT TTG TCA GGT GTG GGG A 3' |
| 7 | *CIZ1* exon 6 forward | 5' TCT TCT CAG ACA ATG CCT GTG G 3' |
| 8 | *CIZ1* exon 7 reverse | 5' GCA GGG CGG TAA ATC TTG G 3' |
| 9 | *CIZ1* exon 11 forward | 5' GGA GAT CCA GCA CAT GAG CC 3' |
| 10 | *CIZ1* exon 12 reverse | 5' CAG GTC CCC CAT GTA GTA GAG CT 3' |
| 11 | *CIZ1* exon 15 forward | 5' ACC TAC AGC CCC AAT ACT GCA TAT 3' |
| 12 | *CIZ1* exon 16 reverse | 5' AGA GCT GTG CCC CTG AGT TG 3' |
| 13 | *CYPA* forward | 5' TTT CAT CTG CAC TGC CAA GAC T 3' |
| 14 | *CYPA* reverse | 5' TTC ATG CCT TCT TTC ACT TTG C 3' |
| 15 | *MYC* forward | 5' GCC ACG TCT CCA CAC ATC AG 3' |
| 16 | *MYC* reverse | 5' TCT TGG CAG CAG GAT AGT CCT T 3' |
|  |  |  |
| **B) Taqman primers & probes** | **Description** | **Sequence** |
| 17 | *CIZ1* exon 3 forward | 5' CTC CAT GCT GCA GAG AGC TT 3' |
| 18 | *CIZ1* exon 5 reverse | 5' GGC CTG GGG AAA GAA CTG TT 3' |
| 19 | *CIZ1* Δ4 probe | 5' FAM-CAG CAG TTG CAA GGT AAC CTC CGA-TAMRA 3' |
| 20 | *CIZ1* exon 6 forward | 5' TGC CTG TGG AAG ACA AGT CA 3' |
| 21 | *CIZ1* exon 7 reverse | 5' TGC TGG AGT GCG TTT TTC CT 3' |
| 22 | *CIZ1* exon 7 probe | 5' JOE CCC TGC CCA GAG GAC ATC GCC-BH 3' |
| 23 | *CIZ1* exon 7 forward | 5' CTG CCA GCA AAG AGA TTG AG 3' |
| 24 | *CIZ1* exon 13 reverse | 5' TGC GAG GGG TTT TGA AGT AG 3' |
| 25 | *CIZ1* f-variant probe | 5' FAM-CAG GGC AGT TAC AGG ACA CAG GAC-TAMRA 3' |
|  |  |  |
| **D) PCR primers** | **Description** | **Sequence** |
| 26 | *CIZ1* exon 1d forward | 5' GCG ACT TGA GCG TTG AG 3' |
| 27 | *CIZ1* exon 5 forward | 5' CTC CTC CTC TAC CAC CCC C 3' |
| 28 | *CIZ1* exon 13 reverse | 5' CAG AAG GGT CGC AAG GAT TGT 3' |
| 29 | *CIZ1* exon 16 reverse | 5' AGA GCT GTG CCC CTG AGT TG 3' |

**References**

1. Greaves EA, Copeland NA, Coverley D, Ainscough JF. Cancer-associated variant expression and interaction of CIZ1 with cyclin A1 in differentiating male germ cells. Journal of cell science 2012; 125:2466-77.

2. Warder DE, Keherly MJ. Ciz1, Cip1 interacting zinc finger protein 1 binds the consensus DNA sequence ARYSR(0-2)YYAC. J Biomed Sci 2003; 10:406-17.

3. Rahman F, Ainscough JF, Copeland N, Coverley D. Cancer-associated missplicing of exon 4 influences the subnuclear distribution of the DNA replication factor CIZ1. Hum Mutat 2007; 28:993-1004.

4. Rahman FA, Aziz N, Coverley D. Differential detection of alternatively spliced variants of Ciz1 in normal and cancer cells using a custom exon-junction microarray. BMC Cancer 2010; 10:482.

5. Dahmcke CM, Buchmann-Moller S, Jensen NA, Mitchelmore C. Altered splicing in exon 8 of the DNA replication factor CIZ1 affects subnuclear distribution and is associated with Alzheimer's disease. Mol Cell Neurosci 2008; 38:589-94.

6. Higgins G, Roper KM, Watson IJ, Blackhall FH, Rom WN, Pass HI, Ainscough JF, Coverley D. Variant Ciz1 is a circulating biomarker for early-stage lung cancer. Proc Natl Acad Sci U S A 2012; 109:E3128-35.

7. Coverley D, Marr J, Ainscough J. Ciz1 promotes mammalian DNA replication. Journal of cell science 2005; 118:101-12.

8. Ainscough JF, Rahman FA, Sercombe H, Sedo A, Gerlach B, Coverley D. C-terminal domains deliver the DNA replication factor Ciz1 to the nuclear matrix. Journal of cell science 2007; 120:115-24.
